# Trypanin Disruption Affects the Motility and Infectivity of the Protozoan *Trypanosoma cruzi*

**DOI:** 10.3389/fcimb.2021.807236

**Published:** 2022-01-07

**Authors:** Jose L. Saenz-Garcia, Beatriz S. Borges, Normanda Souza-Melo, Luiz V. Machado, Juliana S. Miranda, Lisandro Alfonso Pacheco-Lugo, Nilmar S. Moretti, Richard Wheleer, Lia C. Soares Medeiros, Wanderson D. DaRocha

**Affiliations:** ^1^ Laboratório de Genômica Funcional de Parasitos (GFP), Universidade Federal de Paraná, Curitiba, Brazil; ^2^ Laboratório de Biologia Celular, Instituto Carlos Chagas, Fundação Oswaldo Cruz (Fiocruz), Curitiba, Brazil; ^3^ Laboratório de Biologia Molecular de Patógenos (LBMP), Departamento de Microbiologia, Imunologia e Parasitologia, Escola Paulista de Medicina, Universidade Federal de São Paulo, São Paulo, Brazil; ^4^ Laboratório de Ultraestrutura Hertha Mayer, Universidade Federal do Rio de Janeiro (UFRJ), Rio de Janeiro, Brazil; ^5^ Facultad de Ciencias Básicas y Biomédicas, Universidad Simón Bolívar, Barranquilla, Colombia; ^6^ Nuffield Department of Medicine, University of Oxford, Oxford, United Kingdom

**Keywords:** *Trypanosoma cruzi* (*T. cruzi*), trypanin, motility, detached flagellum, metacyclogenesis, CRISPR-Cas9, SaCas9

## Abstract

The flagellum of Trypanosomatids is an organelle that contributes to multiple functions, including motility, cell division, and host–pathogen interaction. Trypanin was first described in *Trypanosoma brucei* and is part of the dynein regulatory complex. *Tb*Trypanin knockdown parasites showed motility defects in procyclic forms; however, silencing in bloodstream forms was lethal. Since *Tb*Trypanin mutants show drastic phenotypic changes in mammalian stages, we decided to evaluate if the *Trypanosoma cru*zi ortholog plays a similar role by using the CRISPR-Cas9 system to generate null mutants. A ribonucleoprotein complex of SaCas9 and sgRNA plus donor oligonucleotide were used to edit both alleles of *Tc*Trypanin without any selectable marker. *Tc*Trypanin −/− epimastigotes showed a lower growth rate, partially detached flagella, normal numbers of nuclei and kinetoplasts, and motility defects such as reduced displacement and speed and increased tumbling propensity. The epimastigote mutant also showed decreased efficiency of *in-vitro* metacyclogenesis. Mutant parasites were able to complete the entire life cycle *in vitro*; however, they showed a reduction in their infection capacity compared with WT and addback cultures. Our data show that *T. cruzi* life cycle stages have differing sensitivities to *Tc*Trypanin deletion. In conclusion, additional work is needed to dissect the motility components of *T. cruzi* and to identify essential molecules for mammalian stages.

## Introduction

*Trypanosoma cruzi* is a protozoan parasite of the Trypanosomatidae family, which bears a group of early diverging eukaryotes. This parasite is the etiological agent of Chagas disease, a potentially life-threatening illness that affects about 6–8 million people around the world (Mills, 2020). *Trypanosoma cruzi* epimastigotes (a replicative form) differentiate into metacyclic trypomastigotes (MTs), the infective and non-replicative forms in triatomine insects. MTs are released within insect feces and, once introduced into a host, can invade almost all nucleated cells. Once inside the host cells, they differentiate into amastigotes, a replicative form containing a short flagellum. After a period of multiplication intracellularly, the amastigote forms transform into bloodstream trypomastigotes; these cells egress the host cells, cross the extracellular matrix, and swim in a crowded and viscous environment (blood) to be able to reach different cell types of the mammalian organism (Ferri and Edreira, 2021). The most studied *T. cruzi* virulence factors are surface proteins, such as gp82, gp63, trans sialidase, and MASP (Bartholomeu et al., 2009; Belew et al., 2017; de Castro Neto et al., 2021). The contribution of parasite motility in this physiological process remains to be elucidated.

*Trypanosoma brucei* and *Leishmania mexicana* parasite motility has been explored extensively compared with *T. cruzi*. To understand *T. brucei* parasite motility, 41 genes were selected based on conserved flagellum genes among other motile eukaryotic organisms and silenced using RNAi approaches. This strategy allowed the identification of new components of motile flagella and the characterization of the phenotype of the mutant based on the severity of the motility defects (Baron et al., 2007). A similar approach was used to characterize 20 new proteins associated with the *T. brucei* paraflagellar rod identified through proteomics (Portman et al., 2009). Using CRISPR/Cas9-assisted gene deletion, Beneke et al. dissected the components of *L. mexicana* flagellum proteome (Beneke et al., 2019). The authors obtained 56 mutants to flagellum proteins with altered swimming speed and morphological defect phenotypes. Interesting, some of the mutants were unable to develop in the insect vector, as observed by infection assays (Beneke et al., 2019).

Unlike *T. brucei*, *T. cruzi* lacks RNAi machinery (DaRocha et al., 2004a), but recent advances on CRISPR/Cas9 technology were adapted to dissect the gene function in this organism (Peng et al., 2014; Lander et al., 2015; Soares Medeiros et al., 2017; Burle-Caldas et al., 2018; Chiurillo and Lander, 2021). Despite these advances in the genetic manipulation methodologies in *T. cruzi*, only few flagellar components, gp72, PFR-1, and PFR-2, were characterized (Cooper et al., 1993; Lander et al., 2015). The lack of gp72 caused detached flagella and reduced metacyclogenesis. Though they were able to infect host cells in culture, they were not able to generate bloodstream trypomastigotes; instead, only amastigote forms were released (de Jesus et al., 1993; Cooper et al., 1993). The multicopy genes PFR-1 and PFR-2 were edited using CRISPR/Cas9 technology, and the null mutant also showed a detached flagellum, and they were unable to build the paraflagellar rod structure as shown by transmission microscopy (Lander et al., 2015).

*Trypanosoma cruzi* and *T. brucei* have conserved flagellar structures, such as an axoneme with 9 + 2 microtubules, and kinetoplastid-specific extra-axonemal structure called paraflagellar rod (PFR). The PFR and axoneme are surrounded by the membrane flagellum and the PFR is linked to microtubules 4 to 7 of the axoneme similar to that in *T. brucei* (De Souza, 2002; Langousis and Hill, 2014). Similar to other Trypanosomatidae, the flagellum beating in *T. cruzi* starts at the tip and propagates to the base. The distance and speed vary cell to cell, with persisting and tumbling modes of motility, with some parasites presenting an intermediate state. This tumbling period seems important for changes in directionality (Ballesteros-Rodea et al., 2012; Walker and Wheeler, 2019). Axoneme structure is necessary for motility in trypanosomatids (Langousis and Hill, 2014). Dynein motors drive the sliding of the microtubules and flagellum beating is produced by the temporal–spatial control of dynein conformations (Lin and Nicastro, 2018). The regulation of dynein conformations requires the nexin–dynein regulator complex (NDRC), where one of the most studied component in trypanosomatids is the protein Trypanin (Ralston and Hill, 2006; Kabututu et al., 2010), the orthologs of which are variously called N-DRC4, GAS8, GAS11, and PF2.

Trypanin was well-characterized in *T. brucei*, an ~54-kDa protein conserved among other trypanosomatids and found mainly in the cytoskeletal fraction (Hill et al., 2000). RNAi targeting of *Tb*Trypanin in procyclic forms causes an uncoordinated flagellar beat and non-directional motility, giving a tumbling motion (Hutchings et al., 2002). *Tb*Trypanin is essential in *T. brucei* bloodstream forms as observed by RNAi experiments, suggesting that perhaps flagellum beating contributes to separating subpellicular microtubules and thus cytokinesis initiation (Ralston and Hill, 2006). Later genetic analysis of other motile components of the flagellum confirmed that normal motility is required for cytokinesis (Broadhead et al., 2006; Ralston et al., 2006).

Later analysis of the *Chlamydomonas* insertional mutagenesis clone pf2 with a defect in motility revealed mutation of *Tb*Trypanin ortholog disrupted N-DRC confirmed by electron microscopy. *pf2* mutants showed defects in the 96-nm repeat of the flagellar axoneme. The N-DRC is a complex of proteins that responds to signals from radial spoke proteins interacting with the central pair complex, coordinating dynein motor activity temporally and spatially and commonly associated with inner arm dyneins (Rupp and Porter, 2003). In mammals, the Trypanin homolog is called GAS11 (growth arrest specific 11) in humans and GAS8 in mice. The finding that GAS11 is expressed in cells without a motile cilium and is associated with the cytoskeleton suggests that, in addition to its suspected role in the regulation of axonemal dynein, it also participates in microtubule/dynein-dependent processes outside the axoneme (Bekker et al., 2007; Colantonio et al., 2006; Evron et al., 2011).

In this work, we characterized *T. cruzi* Trypanin by generating null mutants using CRISPR-Cas9 methodology. We found that *Tc*Trypanin −/− mutants have several phenotypic changes including reduced epimastigote growth and differentiation into metacyclic trypomastigotes, increased ratio of parasites with detached flagellum, motility defects, and reduced infection capacity.

## Material and Methods

### Parasite Maintenance and Growth Curve

*Trypanosoma cruzi* epimastigotes from the Dm28c clone were cultured in a liver infusion tryptose (LIT) medium supplemented with heat-inactivated fetal bovine serum (10%), hemin, and penicillin/streptomycin (Camargo, 1964). MTs were obtained using the protocol described by Contreras et al. (1985). Briefly, epimastigote cultures at the stationary phase were centrifuged at 3,000×*g* and the cell pellets were washed with 1× PBS, then 5 × 10^8^ parasites were resuspended in 1 ml of triatomine artificial urine (TAU, 190 mM NaCl, 17 mM KCl, 8 mM sodium phosphate buffer, 2 mM MgCl_2_, 2 mM CaCl_2_, pH 6.0) and incubated for 2 h at 28°C. After that, the culture was diluted 100-fold in TAU3AAG medium (TAU supplemented with 50 mM sodium glutamate, 10 mM L-proline, 2 mM sodium aspartate, and 10 mM glucose) and incubated for 72 h at 28°C. Then, we calculated the MT yield as previously reported (Balcazar et al., 2017) with minor change by using horse serum instead of human serum. For the growth curve analysis of *Tc*Trypanin −/− mutants and wild-type epimastigotes, log-phase cultures were diluted to 1 × 10^5^ parasites/ml and the parasite multiplication was quantified daily using a Neubauer chamber. Parasite cultures reaching the stationary phase were diluted 10-fold for counting.

To do differential counting of nuclei and kinetoplast per cell, logarithmic phase parasites were DAPI stained as follows: 5 × 10^6^ parasites were harvested and cells were pelleted at 3,000×*g* for 5 min. The cells were washed twice with PBS, and the parasites were coated on slides with Fluoromount-G™ Mounting Medium with DAPI (Thermo Fisher). At least 100 random parasites for each cell line were analyzed using fluorescence microscopy.

### Cell Cycle Analysis

Epimastigote forms at logarithmic phase (5–6 × 10^6^ parasites/ml) were fixed with 70% methanol for 16 h, washed twice with PBS, resuspended in 100 µl of 2× PI solution (3.4 mM Tris–HCl, 10 mM NaCl, propidium iodide 30 µg/ml, and RNAse 100 µg/ml), and added to 100 µl of PBS. These samples were analyzed by flow cytometry using the FACSCanto II equipment, and data were analyzed using the FlowJo 7.6 version software.

### Bioinformatics Analysis

For phylogenetic tree analyses, Trypanin ortholog amino acid sequences from representative flagellated organisms were obtained from the TriTrypDB. This included the Trypanin ortholog from *Homo sapiens*, *Mus musculus*, and *Danio rerio*. The accession number of each sequence and multiple alignment data are found in **Supplementary Figure 1**. The sequences were analyzed using the SeaView v. 5.0.4 software (Gouy et al., 2010) setting BioNJ with bootstrap of 1,000 replicates.

### Plasmid Construction and Single Guide RNA Transcription

Single guide RNA (sgRNA) design to target *Tc*Trypanin (C4B63_48g99) was performed using EuPaGDT (http://grna.ctegd.uga.edu/), selecting *T. cruzi* Dm28c (TriTryDB-28) as a reference genome and the Cas9 nuclease from *Staphylococcus aureus* (*Sa*Cas9) (Peng and Tarleton, 2015). To ensure that the sgRNA could be used to target other *T. cruzi* strains, a BlastN search was done using 50 nt (25 bases upstream of the SaCas9 cleavage site plus 25 downstream) as query, against all available genomes at TriTrypDB. The BlastN output was manually curated.

For sgRNA *in-vitro* transcription, we constructed a plasmid containing a T7 promoter, the SaCas9 sgRNA scaffold, and Hepatitis Delta Virus ribozyme (HDV), which we named pT7-SaCas9sgRNA-*Bsa*I (**Supplementary Figure 2A**). In this plasmid, the specific region of the sgRNA can be replaced by digestion with *Bsa*I followed by cloning of annealed oligonucleotides. The sgRNA-Trypa442 template was created by oligonucleotide annealing of sgRNA-Trypa442-Plus (ATAGGAGAGTCATGAGATGCGGATT) and sgRNA-Trypa442-Minus (AAACAATCCGCATCTCATGACTCTC) and cloning in the *Bsa*I sites of pT7-sgRNA scaffold-HDV, pT7-SaCas9-sgRNA-Trypa442 (**Supplementary Figure 2B**). pT7-SaCas9-sgRNA-Trypa442 was used as a template for the sgRNA *in-vitro* transcription using MEGAscript™ T7 Transcription Kit (Thermo Scientific), following the instructions of the manufacturer. sgRNA quantity and quality were confirmed by Nanodrop™ quantification and 2% agarose gel electrophoresis.

To restore *Tc*Trypanin expression, the full-length coding sequence without the stop codon of *Tc*Trypanin was PCR amplified with Trypa-For-*Xba*I (5′-AAATCTAGAATGCCACCAAAGGCGGTTCGTG-3′) and Trypa-Rev-*Bam*HI (5′-AAAGGATCCCGACAATTCCCGCCGTCAGAAA-3′) oligonucleotides, using genomic DNA from the Dm28c clone as a template. The PCR product was digested with *Xba*I/*Bam*HI and cloned at the same restriction sites in the plasmids pTREX-Amastin::GFP-Neo (Cruz et al., 2012) and pTREX-Amastin::HA-Hygro (unpublished results) replacing the delta-amastin coding sequence. The resulting vectors allow the expression of *Tc*Trypanin fused to GFP or HA tag. These plasmids were transfected by electroporation using the Amaxa Nucleofector, and the G418-resistant population was obtained as described by DaRocha et al. (2004b) and Pacheco-Lugo et al. (2017).

### CRISPR/Cas9 Editing and RFLP Genotyping

Wild-type epimastigotes of Dm28c were chosen to obtain mutant parasites with both alleles of *Tc*Trypanin edited. We performed the protocol described by Soares Medeiros et al. (2017) and Burle-Caldas et al. (2018). Briefly, 5 × 10^6^ log-phase parasites were electroporated with 20 µg of affinity-purified SaCas9 recombinant protein, 10 µg of sgRNA, and 30 µg of single-stranded DNA (ssDNA) donor. We electroporated epimastigote cultures twice with an interval of 7 days between each electroporation, following the electroporation conditions previously described (Pacheco-Lugo et al., 2017). After 48 h of each electroporation, the genomic DNA (gDNA) was isolated using PureLink™ Genomic DNA Mini Kit (Thermo Fisher) from part of the culture to confirm the gene disruption, by PCR reactions using oligonucleotides that allow the amplification of the entire CDS of *Tc*Trypanin. The PCR product was purified, digested with *Bam*HI enzyme, and electrophoresed in 1% agarose gel. *Tc*Trypanin −/− amplicon with Trypa-For-*Xba*I and Trypa-Rev-*Bam*HI primers (described above) is 1,402 bp long and, after *Bam*HI restriction, generates two bands (926 and 428 bp) detected on agarose gel. The resulting restriction digested fragments (restriction fragment length polymorphism—RFLP) was detected by ethidium bromide staining, and visualization was done with the UVP Bioimaging system.

After confirmation of gene editing, transfected parasites were cloned by limiting dilution in a 96-well ELISA plate to obtain clonal populations. To improve parasite growth during the cloning step, 90 μl of conditioned medium (media from the log-phase culture were recovered by centrifugation followed by supernatant filtering) was added to each well, and the plate was maintained in a wet chamber at 28°C and monitored each week for clone growth. One month later, we collected several clones and tested them with RFLP, as described above, to confirm the *Tc*Trypanin null mutant clones (*Tc*Trypanin −/−).

### Video Microscopy

Epimastigotes from the axenic culture of *Tc*Trypanin −/−, *Tc*Trypanin-addback (AB), and WT parasite were collected and counted in a Neubauer chamber. Ten microliters of cell culture at a density of 5 × 10^6^–1 × 10^7^ parasites/ml of the cell was placed on a microscope slide and filmed for at least 30 s at 5 frames per second using a Leica AF6000 Modular System microscope at ×20 magnification using darkfield illumination. Cell swimming was analyzed as previously described (Wheleer, 2017). Briefly, cells were identified in each frame of the darkfield image using a maxima finding algorithm and then cell swimming paths were generated by connecting cell locations based on their movement over the preceding two frames. Mean swimming speed was calculated from these swimming paths.

### Immunofluorescence and Localization of GFP-Tagged *Tc*Trypanin

Cell cultures of *Tc*Trypanin −/−, *Tc*Trypanin −/− carrying pTREX *Tc*Trypanin::HA, and wild type were fixed with 4% paraformaldehyde and coated on poly-lysine coverslips for 20 min. Coverslips were washed with PBS and the cells permeabilized with PBS + 0.1% Triton X-100 and then blocked with 3% (m/v) BSA for 1 h at room temperature (RT). The parasites were incubated with a dilution 1:100 of the monoclonal antibody 2F6 (mAb 2F6), which recognizes a flagellar protein of ~70 kDa (Ramos et al., 2011), for 16 h at 4°C, washed three times with PBS + 0.05% Tween-20, and incubated with the secondary antibody anti-mouse conjugated with Alexa-488 (1:500). The coverslips were washed three times, mounted on a microscope slide, and analyzed by confocal microscopy using a Nikon equipment (confocal microscope A1R multiphoton). Parasites *Tc*Trypanin −/− (pTREX-*Tc*Trypanin::GFP), WT (pTREX-*Tc*Trypanin::GFP), and WT (pTREX-GFP) were fixed with paraformaldehyde 4%, coated on poly-lysine coverslips for 20 min, mounted with Fluoromount-G™ Mounting Medium with DAPI (Thermo Fisher), and visualized in a Nikon confocal microscope.

### Cytoskeleton Preparation

Epimastigotes (5 × 10^6^) of *Tc*Trypanin −/− (pTREX-*Tc*Trypanin::GFP), WT (pTREX-*Tc*Trypanin::GFP), and WT (pTREX-GFP) were coated on poly-lysine coverslips and incubated for 20 min at RT. To preserve the parasite cytoskeleton, 40 μl of cold PEME (100 mM PIPES, 1 mM MgSO_4_, 0.1 mM EDTA, 2 mM EGTA, pH 6.9) + Triton X-100 (1% v/v) was added and incubated for 10 s and washed twice with PBS Gadelha et al., 2005. Then, the parasites were fixed with 4% paraformaldehyde for 10 min at RT, washed with PBS, and resuspended in 200 μl of PBS. The coated coverslips with fixed parasites were mounted with Fluoromount-G™ Mounting Medium with DAPI (Thermo Fisher) and visualized in a Nikon confocal microscope.

### Western Blot and Cell Fractionation

To extract the epimastigote cytoskeleton in cell suspension, WT Dm28c expressing GFP or *Tc*Trypanin −/− expressing *Tc*Trypanin::GFP cultures was centrifuged at 3,000×g and washed twice with PBS, and the cell pellet was incubated with 50 μl of ice-cold PEME + Triton X-100 (1%) buffer on ice for at least 5 min. The samples were centrifuged at 16,000×*g* for 20 min at 4°C. The pellet (cytoskeleton-enriched fraction) and supernatant fractions were collected in two microtubes. The pellet was washed twice with ice-cold PEME, resuspended in 50 μl of cold PEME, and stored at −20°C until the Western blot assay.

Total cell extracts were obtained by harvesting epimastigotes followed by centrifugation at 3,000×*g*. The cell pellet was washed with PBS and resuspended in SDS-PAGE loading buffer to have 1 × 10^7^ parasites per loading in a polyacrylamide gel. After SDS-PAGE, the proteins were transferred to a PVDF membrane, which was blocked with 5% non-fat milk and incubated with polyclonal anti-GFP (1:1,000) at 4°C for 16 h. After the first antibody incubation, the membrane was washed three times with PBS + Tween 0.05% (v/v) and incubated with anti-rabbit conjugated with peroxidase (1:1,000) at 37°C for 1 h. Finally, the membrane was washed three times with PBS + Tween and antibody recognition was detected using the ECL Chemiluminescence Kit (Thermo Fisher) and X-ray film. Images were acquired exposing X-ray films on the UVP Bioimaging system.

### Cell Infection Assays and Tissue-Cultured Derived Trypomastigote Count

Cultures of LLC-MK2 cells were treated with trypsin (0.05%) (Thermo Fisher) and washed twice with PBS. Cells (4 × 10^4^) were placed in a 24-well plate containing coverslips and cultured in RPMI 1640 (Thermo Fisher) supplemented with 5% fetal bovine serum (FBS) in 5% CO_2_. WT, *Tc*Trypanin −/−, and *Tc*Trypanin −/− AB tissue-cultured derived trypomastigotes (TCTs) were harvested from supernatants from previously infected cultures and counted in a Neubauer chamber. Infection assays were performed using an infection ratio of 10 parasites per LLC-MK2 cell (MOI of 10:1) during 2 or 4 h. After the infection period, cells were washed with 1× PBS three times to remove extracellular forms, and fresh RPMI media supplemented with 5% of FBS was added. After 4 days of infection, the TCT release was analyzed by counting in a Neubauer chamber. TCT releasing was determined by harvesting TCTs 4, 5, 6, and 7 days after infection in the same conditions.

### Scanning and Transmission Electron Microscopy

Log-phase epimastigotes were centrifugated at 3,000×*g* for 5 min and washed with 1× PBS. Then, the parasites were processed as described by de Almeida et al. (2021). Samples were analyzed using a scanning electron microscope (Jeol JSM-6010 Plus/LA) operating at 20 keV.

## Results

### Phylogenetic Analysis of *Tc*Trypanin

**Figure 1:**
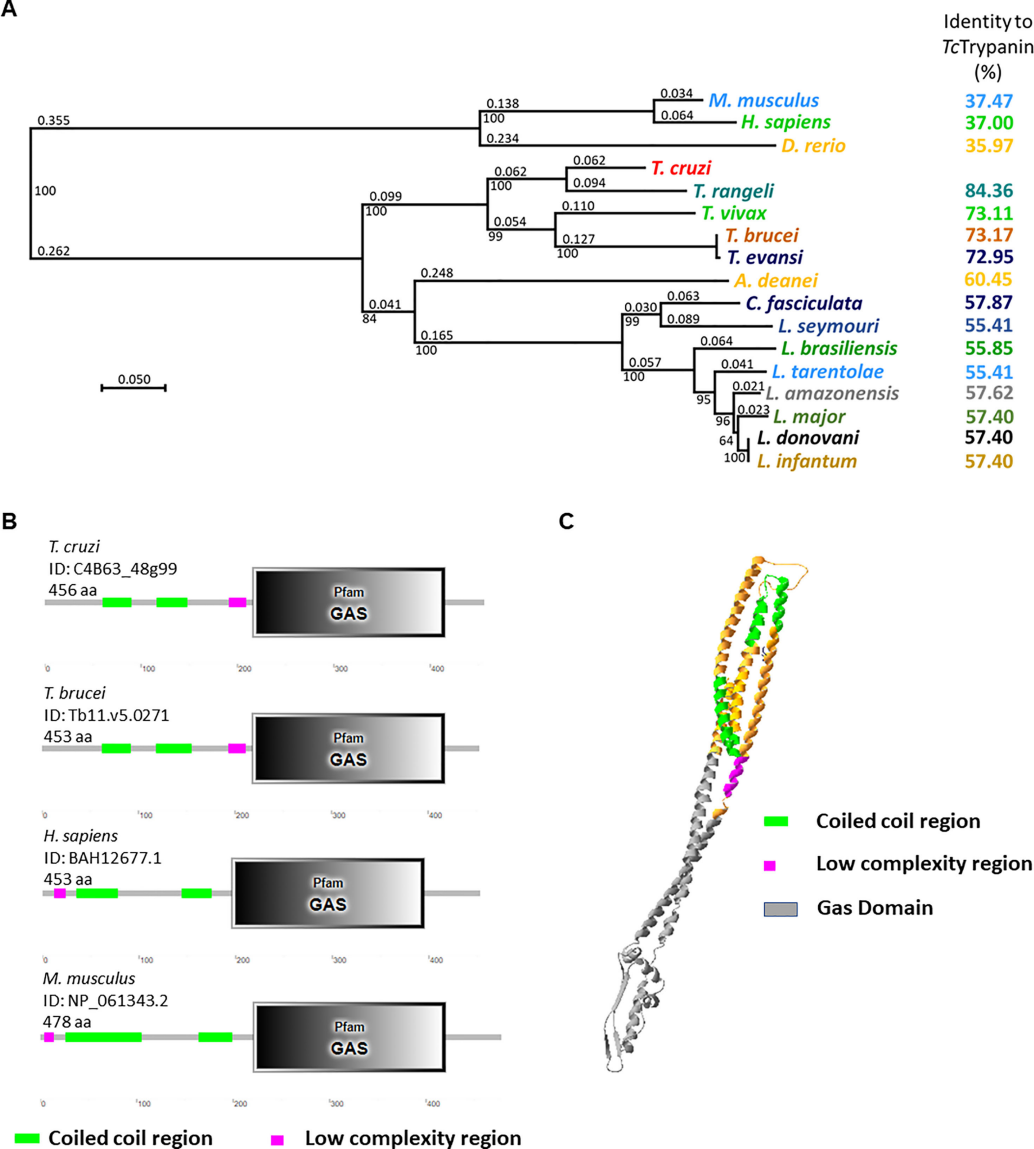
Bioinformatics analysis of *Tc*Trypanin. **(A)** Phylogenetic tree from protein sequences of related tripanosomatids. Amino acid sequences were aligned using SeaView software, and the aligned sequences were used to generate a phylogenetic tree (SeaView 5.4). **(B)** Conserved domains found in *Tc*Trypanin and *Trypanosoma brucei*, human, and mouse orthologs. The bionformatic tool SMART (http://smart.embl-heidelberg.de/) found the conserved GAS domain and coiled coil region at similar regions. **(C)** 3D structure prediction of *Tc*Trypanin protein. The molecular model was generated by the Phyre3 server (Kelley et al., 2015) from the sequence of the *Tc*Trypanin protein from Dm28c (C4B63_48g99). The model shows the predicted tertiary structure that shows higher confidence with colicin IA from *Escherichia coli.* The color scheme corresponds to the features found by SMART analysis.

Trypanin is a highly conserved protein with 37% identity between GAS8 (*Homo sapiens*) sequence and *Tc*Trypanin (*T. cruzi* Dm28c clone—ID: C4B63_48g99). As expected, phylogenetic analysis shows that the *Trypanosoma rangeli* SC58 strain (ID: TRSC58_03641) is the closet related Trypanin among trypanosomatids followed by the *T. brucei* sequence (ID: Tb927.10.6350), sharing 76.32% identity (**Figure 1A**). *Tc*Trypanin presents higher divergence when compared with *Leishmania* species (55.41% to 57.62% identity). For additional evidence of its conservation, we searched for conserved Pfam domains using the SMART software (Letunic, 2004). We found the growth arrest-specific superfamily domain (GAS) (e-value: 6.7 × 10^−60^) starting at position 216 to 415, similar to its orthologs (**Figure 1B**). This domain is present in proteins required for diverse cellular functions such as microtubule organization and cellular division. Flagellated organisms such as *Chlamydomonas* and *T. brucei* have a homolog of GAS8 named N-DRC and *Tb*Trypanin, respectively, and knockdown, disruption, or null mutants showed an altered swimming phenotype. Despite the conservation of *Tc*Trypanin with GAS-related sequences, when we predicted the *Tc*Trypanin 3D structure, it showed higher alignment coverage with colicin IA (structure confidence 98.1) (**Figure 1C**) and myosin II heavy chain (confidence 97.6) (data not shown) likely as it is a long alpha-helix.

It is likely that *Tc*Trypanin forms a coiled-coil tertiary structure, as can be seen in the *Tc*Trypanin model generated by Phyre3 (**Figure 1C**).

### Disruption of *Tc*Trypanin Using the *Sa*Cas9 RNP Complex

**Figure 2:**
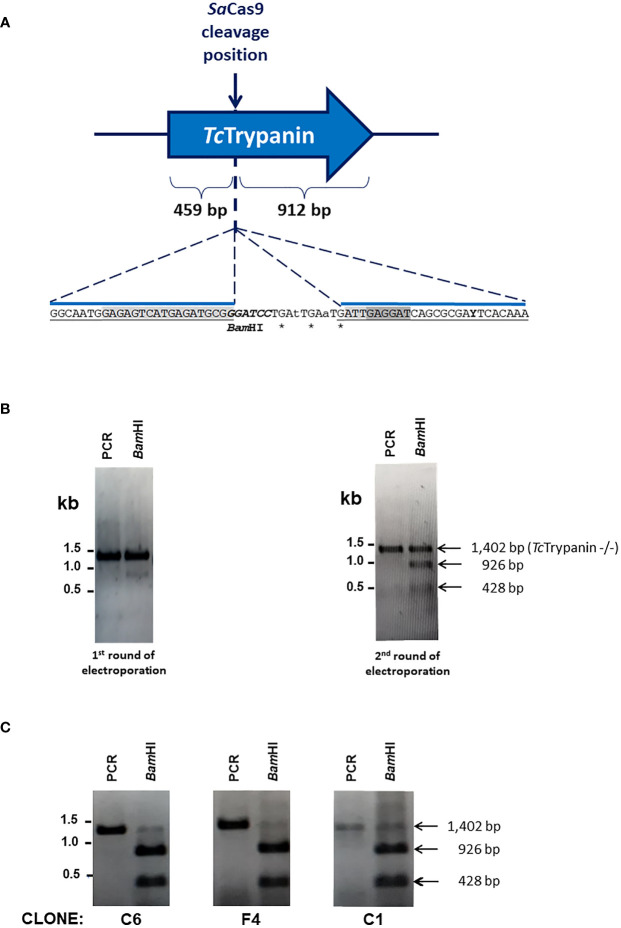
Genome editing of *Tc*Trypanin using the CRISPR/Cas9 RNP complex. **(A)** Schematic representation of the sgRNA targeting site by *Sa*Cas9 RNP complex in the *Tc*Trypanin gene (C4B63_48g99). The RNP complex cleaves right after nucleotide 459 of *Tc*Trypanin coding sequence (CDS length: 1,371 bp). It also represented the insertion point of a *Bam*HI restriction site (italics) to easily track parasite editing through PCR-RFLP and stop codons (asterisks) to ensure coding sequence disruption by homologous recombination using a donor oligonucleotide. The sequence highlighted in light gray in the donor sequence corresponds to the sgRNA target site, and the dark gray sequence is the PAM sequence. **(B)** PCR-RFLP of *Tc*Trypanin showing genome editing of wild-type parasites. The image gels show undigested PCR product (amplicon sizes: WT *Tc*Trypanin = 1,387 bp and *Tc*Trypanin −/− = 1,402 bp) and *Bam*HI-digested (*Bam*HI) PCR product of the full-length ORF of *Tc*Trypanin of two cultures. The left image corresponds to the PCR-RFLP of a mixed population of parasites transfected once with *Sa*Cas9 RNP plus donor sequence. The right gel corresponds to PCR-RFLP of a culture retransfected with RNP complex. **(C)** The PCR-RFLP of individual clones containing both *Tc*Trypanin alleles edited.

To disrupt *Tc*Trypanin, wild-type Dm28c epimastigotes were transfected with a mixture of *Sa*Cas9 recombinant protein preincubated with *in-vitro*-transcribed sgRNA and a donor ssDNA. The donor oligonucleotide has 65 bases corresponding to 3 stop codons at different open reading frames plus a *Bam*HI restriction and 25 bases long homology arms (**Figure 2A**). Since only a small part of the population was edited as detected 3 days after transfection, this population was transfected again, and the new population showed close to 50% of the parasites edited, as detected by RFLP (**Figure 2B**). The *Tc*Trypanin edited population was cloned in a 96-well plate and the clones were checked by RFLP to identify parasites with both *Tc*Trypanin alleles disrupted. Three out of 12 clones were *Tc*Trypanin −/− as shown by RFLP (**Figure 2C**). The use of the ssDNA donor inserting restriction site allowed us to easily identify *Tc*Trypanin −/− clones and occasionally genotype the parasites. Anyhow, the gene disruption was also confirmed by DNA sequencing (**Supplementary Figure 3**).

### *Tc*Trypanin −/− Shows Reduced Parasite Growth and Partially Detached Flagellum in Epimastigotes

**Figure 3:**
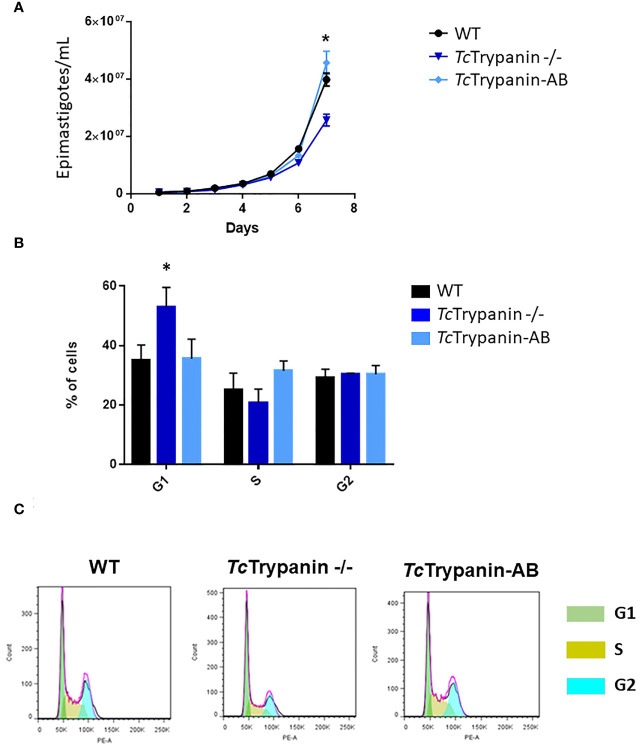
*Tc*Trypanin −/− grows slower than WT and addback cells and presents higher number of cells at the G1 phase. **(A)** Growth curves of WT (Dm28c), *Tc*Trypanin −/−, and *Tc*Trypanin-AB (addback) epimastigotes. The growth curves were performed during 7 days, and the parasites were counted each day (three biological replicates). Asterisk indicates that there is a difference in parasite growth on day 7 (*t*-test, *p* < 0.05). **(B)** Bar graphs show the percentage of cells at each phase of the cell cycle (two biological replicates). The flow cytometry analysis was taken at day 5. Asterisk indicates that G1 cells are more frequent in *Tc*Trypanin −/− cells than in WT cells (*t*-test, *p* < 0.05). **(C)** Flow cytometry chart. Data were analyzed with FlowJo 7.6 version, and analysis of the cell cycle was applied after appropriated gating (this is a representative experiment).

Based on our experience and previously reported work (Santos et al., 2018), epimastigotes from Dm28c clone show log phase (1st to 4th day) reaching 0.65 to 0.85 × 10^7^ parasites/ml, early stationary phase (4th to 7th day) reaching 1.2 × 10^7^ parasites/ml, and stationary phase from day 7 to 10. *Tc*Trypanin disruption is not essential for epimastigote survival, which allowed us to select fully edited clones. However, when we assessed the parasite epimastigote growth profile, *Tc*Trypanin disruption interfered with parasite growth compared with WT cells. The reduction on *Tc*Trypanin −/− growth is small, only becoming significant on the 7th day (**Figure 3A**). To better determine if the phenotypic changes are related to *Tc*Trypanin disruption, we generated an addback culture by overexpressing *Tc*Trypanin fused to HA tag (*Tc*Trypanin::HA). The addback culture, named *Tc*Trypanin-AB, was tested for *Tc*Trypanin::HA expression. As shown in **Supplementary Figure 4**, Western blot and immunofluorescence assay using anti-HA confirm *Tc*Trypanin::HA expression. The growth defect was reversed by *Tc*Trypanin::HA overexpression in *Tc*Trypanin −/− addback culture (**Figure 3A**).

To analyze if this impact on the growth rate could be related to cell cycle progression, log-phase epimastigotes were fixed and stained with propidium iodide. *Tc*Trypanin mutants showed a statistically significant increase in the number of cells in G1 (52.80% ± 6.69%) compared with WT cells (34.96% ± 5.16%) and *Tc*Trypanin −/− AB (35.5% ± 6.64%) (**Figures 3B, C**). DAPI staining analysis of the kinetoplast/nucleus content in epimastigotes showed no evidence for cytokinesis impairment; instead, there is a slight decrease in the number of mitotic cells (2N2K cells) in *Tc*Trypanin −/− epimastigotes (6.5% ± 0.7%) compared with the WT cells (10% ± 1.4%), though it was not statistically significant (**Supplementary Figure 5**). These results demonstrate that *Tc*Trypanin is not crucial for *T. cruzi* cell cycle progression and cytokinesis in the epimastigotes forms.

### Morphology of the *Tc*Trypanin Mutant and Protein Localization

**Figure 4:**
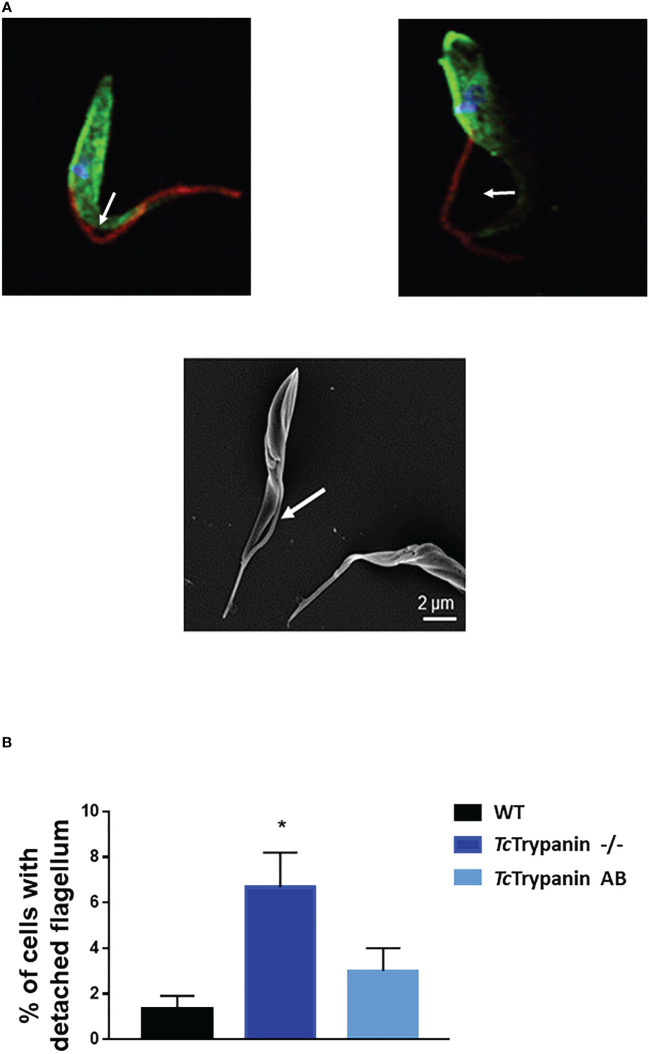
*Tc*Trypanin disruption affects flagellum attachment. **(A)** Immunofluorescence of *Tc*Trypanin disrupted parasites showing detached flagella (white arrows). Mutant parasites (*Tc*Trypanin −/−) were stained with anti-actin (green) and the monoclonal antibody 2F7, a flagellum marker (red) (upper images). The bottom image is a SEM image showing a parasite with a partially detached flagellum. **(B)** Bar graphs plotting the frequency of partially detached flagellum from at least 100 random parasites from immunofluorescence images. The asterisk represents a statistically significant diference between WT and *Tc*Trypanin −/− cells (*t*-test, *p* < 0.05).

Parasite cultures were subjected to immunofluorescence and scanning electron microscopy (SEM) analysis. Similar to what was described for the *Tb*Trypanin knockdown (Ralston et al., 2006), *Tc*Trypanin disruption led to epimastigote flagellum detachment (**Figure 4A**). *Tc*Trypanin −/− culture presented up to 8% of the epimastigotes with partially or totally detached flagella compared with WT parasites (**Figure 4B**).

**Figure 5:**
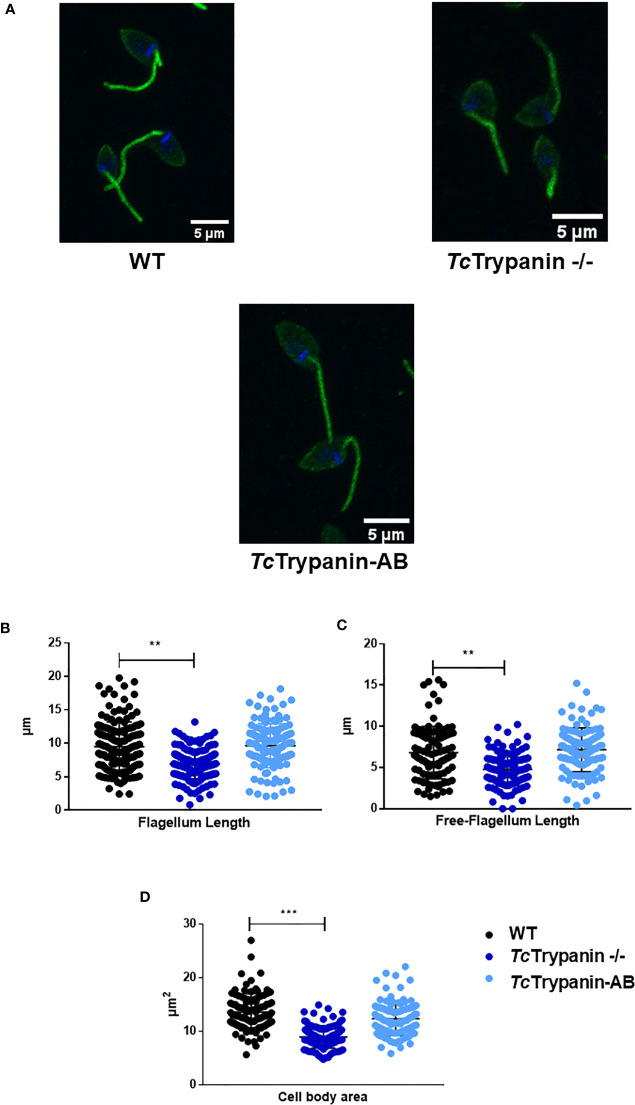
Cell body and flagellum sizes are affected in *Tc*Trypanin −/− mutants. **(A)** Immunofluorescence of epimastigotes with the flagellum marker mAb 2F7. Representative images from parasites immunolabeled with mAb 2F7 (green). **(B–D)** Measurements of flagellum length, free flagellum length, and cell body area. Data of parasite morphology were obtained by measuring the length of the labeled flagellum and cytoplasmic area using ImageJ software. Scatter plot showing data from measures of *N* = 150 parasites stained with monoclonal 2F7 (flagellum marker). The asterisks represent statistically significant difference between WT and *Tc*Trypanin −/−cells (“**” t-test, p< 0.01; “***” p< 0.001).

To determine if *Tc*Trypanin disruption can interfere with parasite size (cell body area) and flagellum length, we performed the measurement of the flagellum from images obtained by immunofluorescence using a monoclonal antibody (clone 2F7) previously described as a flagellum marker (Ramos et al., 2011) and SEM (**Figure 5A** and **Supplementary Figure 6**). The measurement of the flagellum length and cell body area revealed that *Tc*Trypanin −/− parasites are smaller and have a shorter flagellum compared with WT and addback epimastigotes (**Figures 5B**–**D**). WT parasites exhibited a mean flagellum length of 9.51 ± 3.68 µm, while *Tc*Trypanin −/− showed a shorter flagellum (6.89 ± 2.23 µm), and the addback rescued the flagellum length (9.62 ± 3.29 µm) (**Figure 5B**). Body area was calculated to find morphological defects in the cell body. We found that WT cells have a mean area of 13.6 ± 3.43 µm^2^, while *Tc*Trypanin −/− cells are smaller (8.97 ± 2.09 µm^2^), and addback cells recovered their normal size (12.35 ± 3.15 µm^2^) (**Figure 5D**). Similar results were found when SEM images were analyzed (**Supplementary Figure 6**).

**Figure 6:**
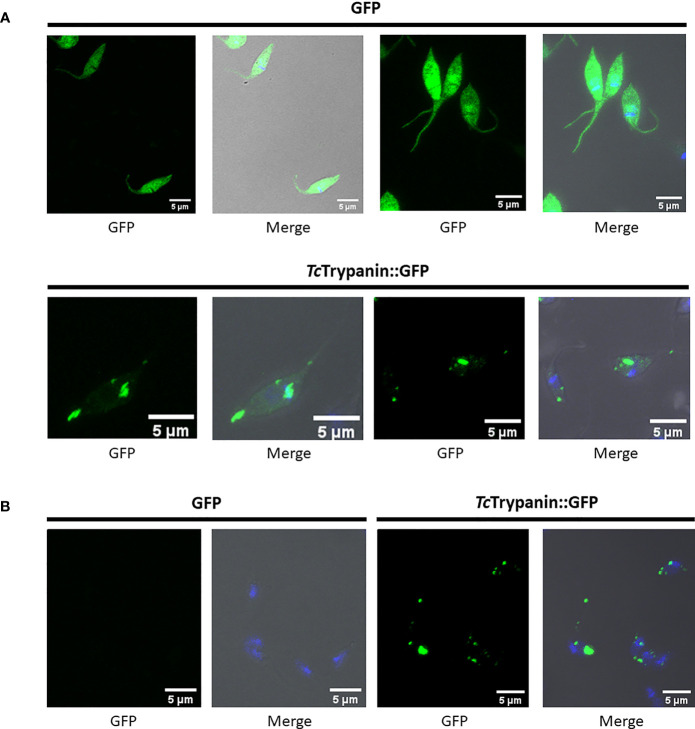
Localization of *Tc*Trypanin::GFP. **(A)** WT parasites were transfected with the plasmid pTREX-*Tc*Trypanin::GFP or pTREX GFP (control plasmid). After G418 resistance selection, the parasites were fixed and analyzed by confocal microscopy. **(B)** The same cultures were processed as described in the *Material and Methods* section to obtain parasite cytoskeleton using PEME + Triton X-100 buffer. For each analyzed field, shown are the GFP channel (left image) and merged images (right image). Merged images correspond to phase-contrast plus GFP (green) and DAPI (blue) channels.

*Tc*Trypanin::GFP localization in fixed parasites (**Figure 6A**) or cytoskeleton preparation (**Figure 6B**) by confocal microscopy showed a distribution unlike *Tb*Trypanin, as described using an epitope tag (Hill et al., 1999) or using polyclonal antibodies (Hill et al., 2000; Hutchings et al., 2002) in *T. brucei*. The GFP-tagged protein localizes to patches of the cortical cytoskeleton (**Figure 6B**). Western blot assay of whole-cell extracts of GFP and *Tc*Trypanin::GFP expressing parasites was conducted. As shown in **Supplementary Figure 7A**, epimastigotes expressed GFP (approximately 26 kDa) and *Tc*Trypanin::GFP (approximately 82 kDa) at the expected size. Furthermore, cell cytoskeleton preparation of parasite suspension followed by Western blot detection of *Tc*Trypanin::GFP confirmed its presence in the cytoskeleton fraction (**Supplementary Figure 7B**), similar to what was described for *Tb*Trypanin in *T. brucei* (Hill et al., 2000). *Tc*Trypanin either has a different subcellular localization to *Tb*Trypanin or N-terminal GFP tagging has disrupted its normal localization. We suspect the latter.

### Motility Is Strongly Impaired in the *Tc*Trypanin Mutant

**Figure 7:**
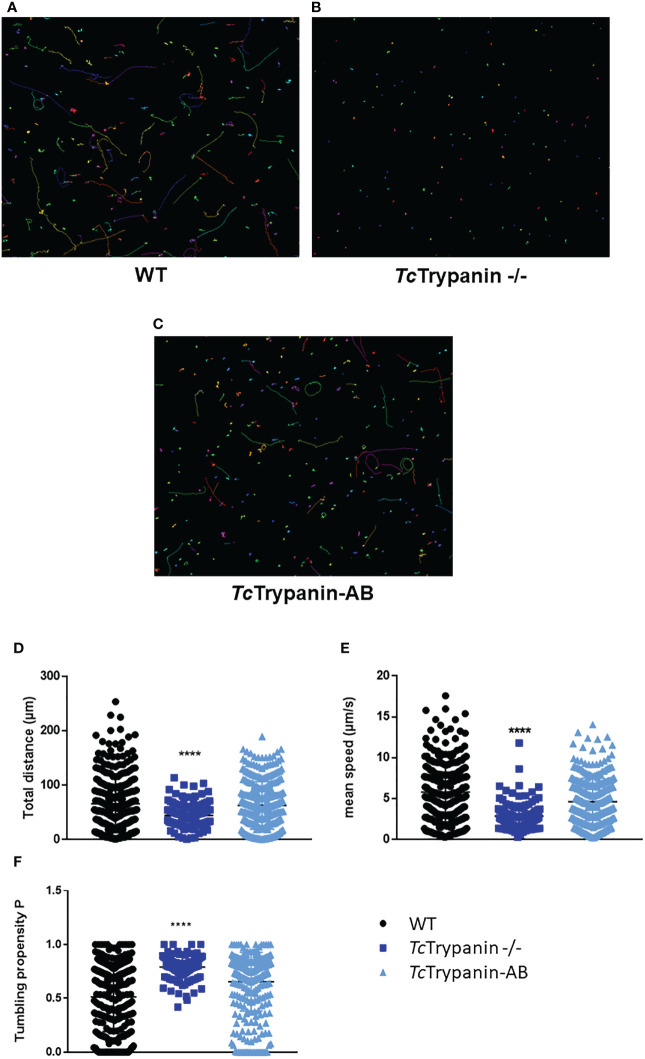
Motility analysis of *Tc*Trypanin −/− by video microscopy. Tracks of WT **(A)**, *Tc*Trypanin −/− **(B)**, and *Tc*Trypanin-AB **(C)** parasites. Epimastigotes were harvested and placed on a microscope slide for video microscopy at ×20 objective. The motility traces of each epimastigote were acquired and converted into images. Different lines or dot colors represent distinct parasites. **(D, E)** Scatter plot of variables from motility analysis. Data from total distance **(D)**, mean speed **(E)**, and tumbling propensity **(F)** were plotted. Two-way ANOVA with multiple comparison and Dunnett test showed a statistical difference with p <0.0001 (four asterisks).

Since *Tb*Trypanin knockdown is required for directional cell motility in *T. brucei* procyclic forms, we decided to determine *Tc*Trypanin −/− epimastigote motility by video microscopy. The images in **Figures 7A**–**C** represent the motility traces of distinct epimastigotes from WT, *Tc*Trypanin −/−, and addback cultures. Wild-type epimastigote cell behavior is similar to previously reported data (Ballesteros-Rodea et al., 2012; Sosa-Hernández et al., 2015), with cell-to-cell variation, where some cells swim productively and some tumble. In *Tc*Trypanin −/−, the motility is strongly reduced with no productively swimming cells (**Figures 7A**–**C**), which we quantified using path length, mean swimming speed, and count of tumbling-like behaviors (**Figures 7D**–**F**). WT epimastigotes showed a higher path length (65 ± 53.09 µm) compared with *Tc*Trypanin −/− (44.27 ± 22 µm), while the *Tc*Trypanin∷HA addback culture (62.87 ± 43.79 µm) recovered the path length defect (**Figure 7D**). The mean speed of the *Tc*Trypanin −/− mutant (2.867 ± 1.59 µm/s) was reduced compared with WT (5.793 ± 3.63 µm/s), with the addback partially recovering swimming speed (4.617 ± 2.68 µm/s) cultures (**Figure 7E**). The *Tc*Trypanin −/− mutants still moved, while not swimming productively, in a tumbling-like behavior. The *Tc*Trypanin −/− parasites presented higher tumbling propensity compared with the other cultures (**Figure 7F**). It is important to highlight that these cells are not immotile despite the short distance traveled, and similar to *T. brucei*, *Tc*Trypanin mutants seem to have a tumbling and twitching motion.

### *Tc*Trypanin Mutants Have Metacyclogenesis and *In-Vitro* Infection Rates Affected

**Figure 8:**
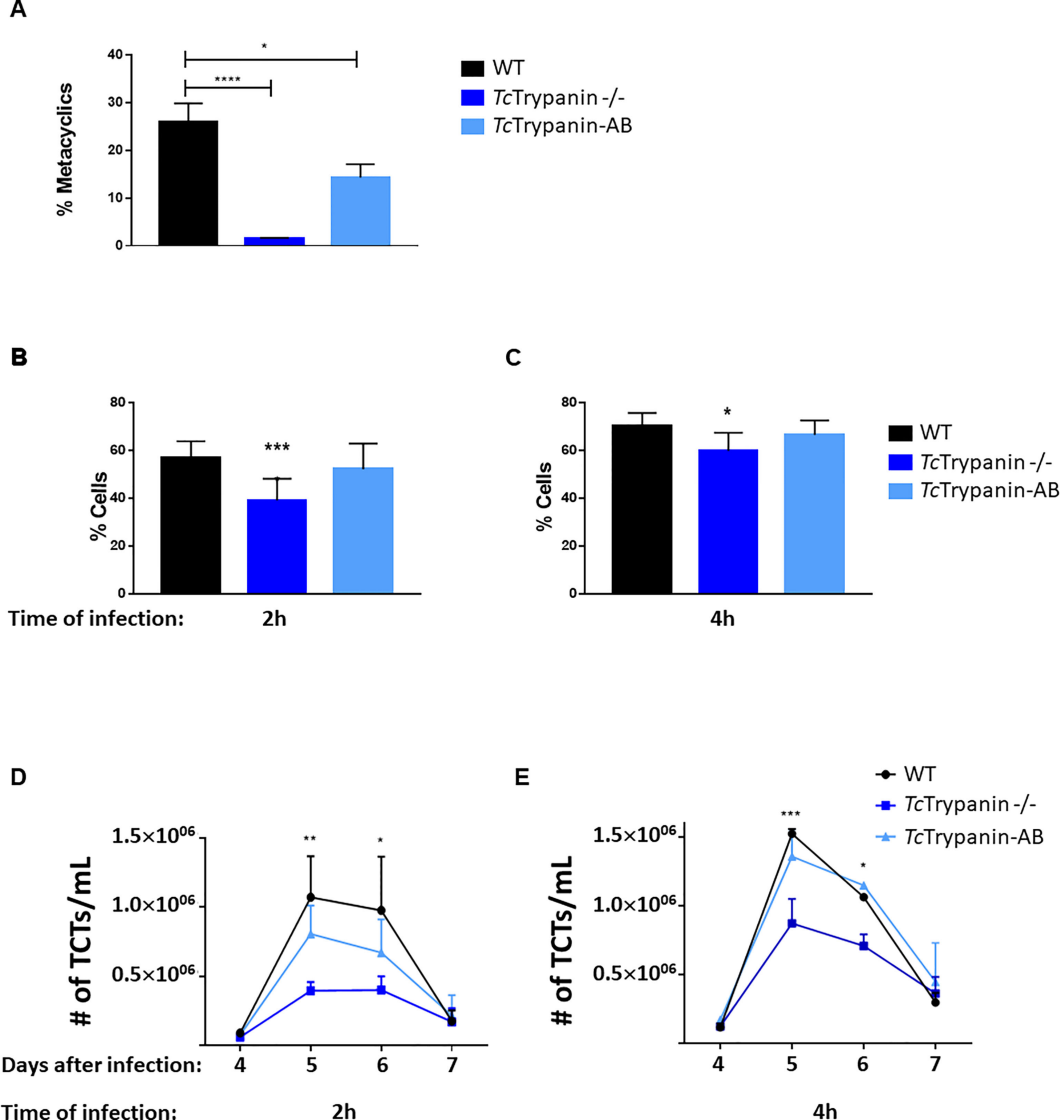
Metacyclogenesis, infection rates, and TCT releasing. **(A)** *Tc*Trypanin disruption affects metacyclogenesis. Epimastigotes from the stationary phase were incubated with TAU medium, and 3 days later, metacyclics were counted. Asterisks indicate statistically significant results (one-way ANOVA, multiple comparison, “*” with p < 0.05, “****” with p < 0.0001). Values from two biological replicates and each of them was run in two technical replicates. **(B, C)** Infection rates of LLC-MK2 cells. Monolayer cultures were challenged with TCTs from WT, *Tc*Trypanin −/−, addback culture. The infections were performed for 2 **(B)** or 4 h **(C)** and Giemsa stained for counting the number of infected cells. This experiment was performed as in three independent replicates with two technical replicates. Asterisk indicates the difference between WT and *Tc*Trypanin −/− (statistical significance was performed in two-way ANOVA, multiple comparison, “*” with p < 0.05, “***” with p < 0.001). **(D, E)** TCT cell releasing dynamic from the supernatant of infected cells. After 4 days of infection, the number of released TCT parasites was counted in a Neubauer chamber. Data were compared with WT and *Tc*Trypanin-AB with two-way ANOVA and the difference was statistically significant as indicated by asterisks (“*” with p < 0.05,“**” with p < 0.01, “***” with p < 0.001). Values are derived from three independent replicates.

To test whether *Tc*Trypanin disruption can somehow affect epimastigote differentiation into metacyclic trypomastigotes *in vitro*, *Tc*Trypanin −/− mutant and wild-type epimastigotes were induced to differentiate for 3 days using TAU + TAU3AAG medium as previously described (Contreras et al., 1985). Wild-type parasites presented 25.76% ± 3.39% of MTs, while *Tc*Trypanin −/− parasites had a much lower number of MTs (1.5% ± 0.14%). The addback of *Tc*Trypanin::HA partially recovered the WT phenotype, by showing 14.28% ± 2.81% (**Figure 8A**). Despite the lower differentiation capacity, the MTs were able to infect LLC-MK2 cells and release TCTs for *in-vitro* infection assays (**Figures 8B**–**D**).

TCTs from a second round of infections were tested on *in-vitro* infection assays as described in the *Material and Methods* section. Mammalian cells were exposed to TCT parasites for 2 or 4 h and washed at least three times. Twelve hours after infection, the cells were stained with Giemsa and counted. After 2 hours of infection, WT showed a higher infection rate of 56.9% ± 6.94% and cells infected with *Tc*Trypanin −/− presented 39.12% ± 9.03%, while addback cultures showed an intermediary rate of 52.08% ± 10.75%. The overall difference between WT and *Tc*Trypanin mutant means was 12.96%, and this difference was statistically significant (*p* < 0.05), while a non-significant difference was detected between WT and addback cultures (**Figure 8B**). When we increased the infection time to 2 h (4 h of infection), WT TCTs were able to invade 70.06% ± 5.56% of cells, while *Tc*Trypanin −/− parasites displayed 59.84% ± 7.55% of infected cells. The overall difference between the means of WT and *Tc*Trypanin −/− was reduced to 10.2% (**Figure 8C**). We suspect this lower infection capacity by the *Tc*Trypanin mutant may be influenced by parasite motility. Performing the same infection conditions, we also evaluated TCT release kinetics after infection per 2 or 4 h (**Figures 8D, E**). All the infected cultures showed a peak of TCT release at day 5, when cells infected with WT parasites during 4 h showed a mean of 1.5 × 10^6^ TCTs/ml released, whereas cells infected with *Tc*Trypanin −/− TCTs released 0.85 × 10^6^ parasites/ml, while again, the addback parasites rescued the TCT release (1.3 × 10^6^ parasites/ml) (**Figure 8E**). In the 2-h cell infection experiment, we detected a similar behavior, where the WT TCT-infected culture released 1 × 10^6^ parasites/ml, *Tc*Trypanin −/− 0.39 × 10^6^, and addback 0.8 × 10^6^ parasites/ml (**Figure 8D**). Taken together, the infection rate (2 and 4 h of TCT incubation) of WT and *Tc*Trypanin mutants somehow correlates with the TCT release, which may suggest that the intracellular stage replication or differentiation may not be affected drastically.

## Discussion

The flagellum of pathogens is a multifunctional organelle frequently associated with parasite infectivity. In *T. cruzi*, the most studied virulence factors are surface proteins. Only recently, with the advance of new genetic editing tools, the initial investigation of motility and infectivity factors has been conducted in this parasite (Arias-del-Angel et al., 2020; Rodríguez et al., 2020). *Trypanosoma cruzi*, like *T. brucei*, needs to travel through the extracellular matrix (a viscous environment) and cross obstacles during a natural infection (Heddergott et al., 2012). Additionally, *T. cruzi* has to attach to cells for receptor recognition (Campetella et al., 2020) and to trigger events for internalization (Maeda et al., 2012). Besides that, the functional characterization of the flagellar component is poorly explored in *T. cruzi*. In this work, we showed for the first time the functional characterization of one axonemal protein of *T. cruzi* and its involvement in motility and infectivity, suggesting the role of motility as an infectivity factor in *T. cruzi.*

To ablate *Tc*Trypanin expression, we failed to replace this gene by selectable markers (NeoR and HygroR) using conventional knockout strategy (unpublished data). However, to disrupt *Tc*Trypanin in *T. cruzi* Dm28c, we performed genome editing with CRISPR/Cas9. For *T. cruzi*, successful editing can be achieved by different methods such as the stable expression of *Sp*Cas9 followed by the transient transfection with *in-vitro*-transcribed sgRNA (Peng et al., 2014; Burle-Caldas et al., 2018; Romagnoli et al., 2018), the stable expression of both *Sp*Cas9 and sgRNA (Lander et al., 2015), the stable expression of T7 RNA polymerase and *Sp*Cas9 followed by the transient transfection of a DNA template for the sgRNA expression directed by the T7 promoter (Costa et al., 2018), and last but not least, the marker free editing by the transfection of the ribonucleoprotein complex (*Sa*Cas9 + sgRNA) protein *Sa*Cas9 (Soares Medeiros et al., 2017; Burle-Caldas et al., 2018). Recombinant protein electroporation was also applied for CRE recombinase delivery to manipulate *T. cruzi* gene expression (Pacheco-Lugo et al., 2020). By applying the *Sa*Cas9 protein delivery, we easily disrupted *Tc*Trypanin using a donor oligonucleotide to insert stop codons plus a restriction site, when compared with the conventional approach. This approach was so powerful that it allowed us to identify mutant parasites a few days after transfection without the use of selectable markers. This reserves selectable markers for additional genetic manipulation steps such as complementation. Currently, a marker-free approach has been used to attenuate *Leishmania major* for vaccine development (Zhang et al., 2020), a requirement for use in preclinical and clinical studies (Karmakar et al., 2021). The *Tc*Trypanin −/− parasite we have developed here are suitable to be tested as a vaccine candidate, since it showed reduced motility and lower infectivity *in vitro*.

*Tc*Trypanin was not essential in insect or mammalian stages. The null mutant had a lower growth rate and an accumulation of cells in G1 in epimastigotes compared with wild-type and addback cultures. Functional analysis of some motile components, including *Tb*Trypanin by mRNA knockdown, showed moderate to severe defects in the cytokinesis of *T. brucei* parasites (Hutchings et al., 2002; Baron et al., 2007). Cytokinesis defects of *Tb*Trypanin knockdown produce a clump of parasites suggesting cytokinesis failure (Ralston et al., 2006); again, we did not observe this kind of phenotype in the *T. cruzi* life cycle stages. This suggests that a motile flagellum has less contribution on *T. cruzi* division, which may be linked with *T. cruzi* having an amastigote life cycle stage. It is important to highlight that *Tc*Trypanin −/− epimastigotes are not immotile and have an active flagellar beating that could be enough for cytokinesis despite its lack of directionality. Whether or not motility is essential for *T. cruzi* cytokinesis might be further explored by deleting genes such as PF16 or another CMF with a severe motility defect (Baron et al., 2007). A previous work with gp72-KO is also informative (de Jesus et al., 1993). In this report, the authors show that gp72 (FLA-1 in *T. brucei*) mutants have detached flagella, and the epimastigote mutants are able to differentiate into infective metacyclic forms (reduced compared with WT). However, the infected cells do not release tissue culture-derived trypomastigotes, only amastigote-like forms with short flagella.

The reduction of cell size was an unexpected result, though the shape of trypanosomatids depends on subpellicular microtubules (SM) (Sinclair et al., 2021), and *T. brucei* cell motility seems to shape SM (Sun et al., 2018). Sun et al. showed that flagellar detachment caused by RNAi of FLA1BP leads to abnormal SM handedness compared with parasites with a normal flagellar wave. Otherwise, differences in cell body size were not reported. This may be due to the transient nature of the RNAi knockdown compared with the permanent disruption of *Tc*Trypanin −/− by CRISPR-Cas9 or differing morphogenesis in *T. brucei*. Flagellum attachment zone proteins (FAZ) form a complex that maintains flagellum attached to the cell body, but that also regulates the cell cycle and coordinate flagellum length, and FLA1BP silencing in *T. brucei* reduces flagellum length (Sun et al., 2012). Hutchings et al. (2002) suggest that *Tb*Trypanin is necessary for normal flagellum attachment. It is unclear whether this is a direct role or an indirect impact of defective motility, but flagellum detachment may contribute to defective morphogenesis in the *Tc*Trypanin −/− mutant.

Our data show that *Tc*Trypanin disruption has a higher impact on TCT infectivity depending on the time of infection (4 vs. 2 h) compared with wild-type parasites, suggesting that motility might be important for parasites to find host cells. Hence, mutant parasites need more time to infect a cell host and will likely be more exposed to the immune system during an *in-vivo* infection. For *T. brucei*, motility impairment leads to higher targeting by the higher clearance of antibodies (Shimogawa et al., 2018). It is possible that the TCTs from *Tc*Trypanin mutant will be less infective on *in-vivo* infection in an animal model. The reduced TCT releasing in *Tc*Trtypanin −/− correlates with fewer infected cells, suggesting that amastigote replication is not affected in the *Tc*Trypanin −/−.

In summary, the *Tc*Trypanin null mutant affected several features of the *T. cruzi* cell biology and life cycle, including epimastigote growth, cell body and flagellum size, flagellum attachment, metacyclogenesis, infection rates, and cell motility. Some of these phenotypic variations were compatible with its ortholog knockdown in *T. brucei.* Despite many similarities with *T. brucei*, the *Tc*Trypanin −/− mutant can go through an entire life cycle. These data reinforce the need for additional studies to understand the functioning of flagellar components in *T. cruzi*.

## Conclusions

We described that *Tc*Trypanin absence causes some motility defects that do not impair cytokinesis in the epimastigote forms. It is also remarkable that these mutants can complete infection cycles despite the reduction in infectivity and metacyclogenesis. This ability to go through the entire life cycle suggests that parasite motility does not affect parasite viability, as seen by the knocking out of proteins related to motility (e.g., gp72/FLA-1 and Trypanin). These data highlight the need for a large-scale functional genomics approach of flagellar components of *T. cruzi*.

## Data Availability Statement

The original contributions presented in the study are included in the article/**Supplementary Material**. Further inquiries can be directed to the corresponding author.

## Author Contributions

JS-G, BB, NS-M, LVM, JSM, LAP-L, LCSM, and RW performed the experiments. JS-G drafted the manuscript. JS-G, NS-M, and WD critically revised the manuscript. All authors contributed to the article and approved the submitted version.

## Funding

The authors were supported by the Fundação Araucária (Grant 044/2017, 48018) (PPSUS-SESA/PR, MS-Decit and Fundação Oswaldo Cruz/Instituto Carlos Chagas), National Counsel of Technological and Scientific Development (CNPq), and CAPES agency (CT-INFRA FINEP, PROEX, and PROAP programs). LM has scholarship from CAPES, and JS-G and WD have scholarships from CNPq. This work was also supported by the Fundação de Amparo à Pesquisa do Estado de São Paulo (FAPESP), grants 2018/09948-0 and 2020/07870-4 to NS-M, and by Conselho Nacional de Desenvolvimento Científico e Tecnológico (CNPq), grants 424729/2018-0 to NSM. We also thank the Wellcome Trust (211075/Z/18/Z) for funding RW research group.

## Conflict of Interest

The authors declare that the research was conducted in the absence of any commercial or financial relationships that could be construed as a potential conflict of interest.

## Publisher’s Note

All claims expressed in this article are solely those of the authors and do not necessarily represent those of their affiliated organizations, or those of the publisher, the editors and the reviewers. Any product that may be evaluated in this article, or claim that may be made by its manufacturer, is not guaranteed or endorsed by the publisher.

## References

[B1] Arias-del-Angel J. A. Santana-Solano J. Santillán M. Manning-Cela R. G. (2020). Motility Patterns of Trypanosoma Cruzi Trypomastigotes Correlate With the Efficiency of Parasite Invasion *In Vitro* . Sci. Rep. 10 (1), 15894. doi: 10.1038/s41598-020-72604-4 32985548 PMC7522242

[B2] Balcazar D. E. Vanrell M. C. Romano P. S. Pereira C. A. Goldbaum F. A. Bonomi H. R. Carrillo C. (2017). The Superfamily Keeps Growing: Identification in Trypanosomatids of RibJ, the First Riboflavin Transporter Family in Protists. PLOS Negl. Trop. Dis. 11 (4), e0005513. doi: 10.1371/journal.pntd.0005513 28406895 PMC5404878

[B3] Ballesteros-Rodea G. Santillán M. Martínez-Calvillo S. Manning-Cela R. (2012). Flagellar Motility of Trypanosoma Cruzi Epimastigotes. J. Biomed. Biotechnol. 2012, 1–9. doi: 10.1155/2012/520380 22287834 PMC3263639

[B4] Baron D. M. Ralston K. S. Kabututu Z. P. Hill K. L. (2007). Functional Genomics in Trypanosoma Brucei Identifies Evolutionarily Conserved Components of Motile Flagella. J. Cell Sci. 120 (3), 478–491. doi: 10.1242/jcs.03352 17227795

[B5] Bartholomeu D. C. Cerqueira G. C. Leão A. C. A. daRocha W. D. Pais F. S. Macedo C.. (2009). Genomic Organization and Expression Profile of the Mucin-Associated Surface Protein (Masp) Family of the Human Pathogen Trypanosoma Cruzi. Nucleic Acids Res. 37 (10), 3407–3417. doi: 10.1093/nar/gkp172 19336417 PMC2691823

[B6] Bekker J. M. Colantonio J. R. Stephens A. D. Clarke W. T. King S. J. Hill K. L.. (2007). Direct Interaction of Gas11 With Microtubules: Implications for the Dynein Regulatory Complex. Cell Motil. Cytoskeleton 64 (6), 461–473. doi: 10.1002/cm.20196 17366626

[B7] Belew A. T. Junqueira C. Rodrigues-Luiz G. F. Valente B. M. Oliveira A. E. R. Polidoro R. B.. (2017). Comparative Transcriptome Profiling of Virulent and Non-Virulent Trypanosoma Cruzi Underlines the Role of Surface Proteins During Infection. PloS Pathog. 13 (12), e1006767. doi: 10.1371/journal.ppat.1006767 29240831 PMC5746284

[B8] Beneke T. Demay F. Hookway E. Ashman N. Jeffery H. Smith J.. (2019). Genetic Dissection of a Leishmania Flagellar Proteome Demonstrates Requirement for Directional Motility in Sand Fly Infections. PloS Pathog. 15 (6), e1007828. doi: 10.1371/journal.ppat.1007828 31242261 PMC6615630

[B9] Broadhead R. Dawe H. R. Farr H. Griffiths S. Hart S. R. Portman N.. (2006). Flagellar Motility Is Required for the Viability of the Bloodstream Trypanosome. Nature 440 (7081), 224–227. doi: 10.1038/nature04541 16525475

[B10] Burle-Caldas G. A. Soares-Simões M. Lemos-Pechnicki L. DaRocha W. D. Teixeira S. M. R. (2018). Assessment of Two CRISPR-Cas9 Genome Editing Protocols for Rapid Generation of Trypanosoma Cruzi Gene Knockout Mutants. Int. J. Parasitol. 48 (8), 591–596. doi: 10.1016/j.ijpara.2018.02.002 29577891

[B11] Camargo E. P. (1964). Growth and Differentiation in Trypanosoma Cruzi. I. Origin of Metacyclic Trypanosomes in Liquid Media. Rev. Do Inst. Med. Trop. Sao Paulo 6, 93–100. 14177814

[B12] Campetella O. Buscaglia C. A. Mucci J. Leguizamón M. S. (2020). Parasite-Host Glycan Interactions During Trypanosoma Cruzi Infection: Trans-Sialidase Rides the Show. Biochim. Biophys. Acta (BBA) - Mol. Basis Dis. 1866 (5), 165692. doi: 10.1016/j.bbadis.2020.165692 PMC7819670 31972227

[B13] Chiurillo M. A. Lander N. (2021). The Long and Winding Road of Reverse Genetics in Trypanosoma Cruzi. Microb. Cell 8 (9), 203–207. doi: 10.15698/mic2021.09.758 34527719 PMC8404153

[B14] Colantonio J. R. Bekker J. M. Kim S. J. Morrissey K. M. Crosbie R. H. Hill K. L. (2006). Expanding the Role of the Dynein Regulatory Complex to Non-Axonemal Functions: Association of GAS11 With the Golgi Apparatus: GAS11 Associates With the Golgi Apparatus. Traffic 7 (5), 538–548. doi: 10.1111/j.1600-0854.2006.00411.x 16643277

[B15] Contreras V. T. Salles J. M. Thomas N. Morel C. M. Goldenberg S. (1985). *In Vitro* Differentiation of Trypanosoma Cruzi Under Chemically Defined Conditions. Mol. Biochem. Parasitol. 16 (3), 315–327. doi: 10.1016/0166-6851(85)90073-8 3903496

[B16] Cooper R. de Jesus A. R. Cross G. A. (1993). Deletion of an Immunodominant Trypanosoma Cruzi Surface Glycoprotein Disrupts Flagellum-Cell Adhesion. J. Cell Biol. 122 (1), 149–156. doi: 10.1083/jcb.122.1.149 8314840 PMC2119612

[B17] Costa F. C. Francisco A. F. Jayawardhana S. Calderano S. G. Lewis M. D. Olmo F.. (2018). Expanding the Toolbox for Trypanosoma Cruzi: A Parasite Line Incorporating a Bioluminescence-Fluorescence Dual Reporter and Streamlined CRISPR/Cas9 Functionality for Rapid *In Vivo* Localisation and Phenotyping. PloS Negl. Trop. Dis. 12 (4), e0006388. doi: 10.1371/journal.pntd.0006388 29608569 PMC5897030

[B18] Cruz M. C. Souza-Melo N. da Silva C. V. DaRocha W. D. Bahia D. Araújo P. R.. (2012). Trypanosoma Cruzi: Role of δ-Amastin on Extracellular Amastigote Cell Invasion and Differentiation. PloS One 7 (12), e51804. doi: 10.1371/journal.pone.0051804 23272170 PMC3525664

[B19] DaRocha W. D. Otsu K. Teixeira S. M. R. Donelson J. E. (2004a). Tests of Cytoplasmic RNA Interference (RNAi) and Construction of a Tetracycline-Inducible T7 Promoter System in Trypanosoma Cruzi. Mol. Biochem. Parasitol. 133 (2), 175–186. doi: 10.1016/j.molbiopara.2003.10.005 14698430

[B20] DaRocha W. D. Silva R. A. Bartholomeu D. C. Pires S. F. Freitas J. M. Macedo A. M.. (2004b). Expression of Exogenous Genes in Trypanosoma Cruzi: Improving Vectors and Electroporation Protocols. Parasitol. Res. 92 (2), 113–120. doi: 10.1007/s00436-003-1004-5 14634799

[B21] de Almeida J. M. Nunes F. O. Ceole L. F. Klimeck T. D. F. da Cruz L. A. Tófoli D.. (2021). Synergistic Effect and Ultrastructural Changes in Trypanosoma Cruzi Caused by Isoobtusilactone A in Short Exposure of Time. PloS One 16 (1), e0245882. doi: 10.1371/journal.pone.0245882 33507972 PMC7842926

[B22] de Castro Neto A. L. da Silveira J. F. Mortara R. A. (2021). Comparative Analysis of Virulence Mechanisms of Trypanosomatids Pathogenic to Humans. Front. Cell. Infect. Microbiol. 11, 669079. doi: 10.3389/fcimb.2021.669079 33937106 PMC8085324

[B23] de Jesus A. R. Cooper R. Espinosa M. Gomes J. E. Garcia E. S. Paul S.. (1993). Gene Deletion Suggests a Role for Trypanosoma Cruzi Surface Glycoprotein GP72 in the Insect and Mammalian Stages of the Life Cycle. J. Cell Sci. 106 ( Pt 4), 1023–1033. doi: 10.1242/jcs.106.4.1023 8126090

[B24] De Souza W. (2002). Basic Cell Biology of Trypanosoma Cruzi. Curr. Pharm. Des. 8 (4), 269–285. doi: 10.2174/1381612023396276 11860366

[B25] Evron T. Philipp M. Lu J. Meloni A. R. Burkhalter M. Chen W.. (2011). Growth Arrest Specific 8 (Gas8) and G Protein-Coupled Receptor Kinase 2 (GRK2) Cooperate in the Control of Smoothened Signaling. J. Biol. Chem. 286 (31), 27676–27686. doi: 10.1074/jbc.M111.234666 21659505 PMC3149358

[B26] Ferri G. Edreira M. M. (2021). All Roads Lead to Cytosol: Trypanosoma Cruzi Multi-Strategic Approach to Invasion. Front. Cell. Infect. Microbiol. 11, 634793. doi: 10.3389/fcimb.2021.634793 33747982 PMC7973469

[B27] Gadelha C. Wickstead B. de Souza W. Gull K. Cunha-e-Silva N. (2005). Cryptic Paraflagellar Rod in Endosymbiont-Containing Kinetoplastid Protozoa. Eukaryot. Cell 4 (3), 516–525. doi: 10.1128/EC.4.3.516-525.2005 15755914 PMC1087800

[B28] Gouy M. Guindon S. Gascuel O. (2010). SeaView Version 4: A Multiplatform Graphical User Interface for Sequence Alignment and Phylogenetic Tree Building. Mol. Biol. Evol. 27 (2), 221–224. doi: 10.1093/molbev/msp259 19854763

[B29] Heddergott N. Krüger T. Babu S. B. Wei A. Stellamanns E. Uppaluri S.. (2012). Trypanosome Motion Represents an Adaptation to the Crowded Environment of the Vertebrate Bloodstream. PloS Pathog. 8 (11), e1003023. doi: 10.1371/journal.ppat.1003023 23166495 PMC3499580

[B30] Hill K. L. Hutchings N. R. Grandgenett P. M. Donelson J. E. (2000). T Lymphocyte-Triggering Factor of African Trypanosomes Is Associated With the Flagellar Fraction of the Cytoskeleton and Represents a New Family of Proteins That Are Present in Several Divergent Eukaryotes. J. Biol. Chem. 275 (50), 39369–39378. doi: 10.1074/jbc.M006907200 10969087

[B31] Hill K. L. Hutchings N. R. Russell D. G. Donelson J. E. (1999). A Novel Protein Targeting Domain Directs Proteins to the Anterior Cytoplasmic Face of the Flagellar Pocket in African Trypanosomes. J. Cell Sci. 112 Pt 18, 3091–3101. doi: 10.1242/jcs.112.18.3091 10462525

[B32] Hutchings N. R. Donelson J. E. Hill K. L. (2002). Trypanin Is a Cytoskeletal Linker Protein and Is Required for Cell Motility in African Trypanosomes. J. Cell Biol. 156 (5), 867–877. doi: 10.1083/jcb.200201036 11864997 PMC2173309

[B33] Kabututu Z. P. Thayer M. Melehani J. H. Hill K. L. (2010). CMF70 Is a Subunit of the Dynein Regulatory Complex. J. Cell Sci. 123 (20), 3587–3595. doi: 10.1242/jcs.073817 20876659 PMC2951471

[B34] Karmakar S. Ismail N. Oliveira F. Oristian J. Zhang W. W. Kaviraj S.. (2021). Preclinical Validation of a Live Attenuated Dermotropic Leishmania Vaccine Against Vector Transmitted Fatal Visceral Leishmaniasis. Commun. Biol. 4 (1), 929. doi: 10.1038/s42003-021-02446-x 34330999 PMC8324786

[B35] Kelley L. A. Mezulis S. Yates C. M. Wass M. N. Sternberg M. J. E. (2015). The Phyre2 Web Portal for Protein Modeling, Prediction and Analysis. Nat. Protoc. 10 (6), 845–858. doi: 10.1038/nprot.2015.053 25950237 PMC5298202

[B36] Lander N. Li Z.-H. Niyogi S. Docampo R. (2015). CRISPR/Cas9-Induced Disruption of Paraflagellar Rod Protein 1 and 2 Genes in Trypanosoma Cruzi Reveals Their Role in Flagellar Attachment. MBio 6 (4), e01012–e01015. doi: 10.1128/mBio.01012-15 26199333 PMC4513075

[B37] Langousis G. Hill K. L. (2014). Motility and More: The Flagellum of Trypanosoma Brucei. Nat. Rev. Microbiol. 12 (7), 505–518. doi: 10.1038/nrmicro3274 24931043 PMC4278896

[B38] Letunic I. (2004). SMART 4.0: Towards Genomic Data Integration. Nucleic Acids Res. 32 (90001), 142D –1 144. doi: 10.1093/nar/gkh088 PMC308822 14681379

[B39] Lin J. Nicastro D. (2018). Asymmetric Distribution and Spatial Switching of Dynein Activity Generates Ciliary Motility. Science 360 (6387), eaar1968. doi: 10.1126/science.aar1968 29700238 PMC6640125

[B40] Maeda F. Y. Cortez C. Yoshida N. (2012). Cell Signaling During Trypanosoma Cruzi Invasion. Front. Immunol. 3, 361. doi: 10.3389/fimmu.2012.00361 23230440 PMC3515895

[B41] Mills R. M. (2020). Chagas Disease: Epidemiology and Barriers to Treatment. Am. J. Med. 133 (11), 1262–1265. doi: 10.1016/j.amjmed.2020.05.022 32592664

[B42] Pacheco-Lugo L. Díaz-Olmos Y. Sáenz-García J. Probst C. M. DaRocha W. D. (2017). Effective Gene Delivery to Trypanosoma Cruzi Epimastigotes Through Nucleofection. Parasitol. Int. 66 (3), 236–239. doi: 10.1016/j.parint.2017.01.019 28137669

[B43] Pacheco-Lugo L. A. Sáenz-García J. L. Díaz-Olmos Y. Netto-Costa R. Brant R. S. C. DaRocha W. D. (2020). CREditing: A Tool for Gene Tuning in Trypanosoma Cruzi. Int. J. Parasitol. 50 (13), 1067–1077. doi: 10.1016/j.ijpara.2020.06.010 32858036

[B44] Peng D. Kurup S. P. Yao P. Y. Minning T. A. Tarleton R. L. (2014). CRISPR-Cas9-Mediated Single-Gene and Gene Family Disruption in Trypanosoma Cruzi. MBio 6 (1), e02097–14. doi: 10.1128/mBio.02097-14 PMC4281920 25550322

[B45] Peng D. Tarleton R. (2015). EuPaGDT: A Web Tool Tailored to Design CRISPR Guide RNAs for Eukaryotic Pathogens. Microb. Genomics 1 (4), e000033. doi: 10.1099/mgen.0.000033 PMC5320623 28348817

[B46] Portman N. Lacomble S. Thomas B. McKean P. G. Gull K. (2009). Combining RNA Interference Mutants and Comparative Proteomics to Identify Protein Components and Dependences in a Eukaryotic Flagellum. J. Biol. Chem. 284 (9), 5610–5619. doi: 10.1074/jbc.M808859200 19074134 PMC2645819

[B47] Ralston K. S. Hill K. L. (2006). Trypanin, a Component of the Flagellar Dynein Regulatory Complex, Is Essential in Bloodstream Form African Trypanosomes. PloS Pathog. 2 (9), e101. doi: 10.1371/journal.ppat.0020101 17009870 PMC1579245

[B48] Ralston K. S. Lerner A. G. Diener D. R. Hill K. L. (2006). Flagellar Motility Contributes to Cytokinesis in Trypanosoma Brucei and Is Modulated by an Evolutionarily Conserved Dynein Regulatory System. Eukaryot. Cell 5 (4), 696–711. doi: 10.1128/EC.5.4.696-711.2006 16607017 PMC1459671

[B49] Ramos T. C. P. Freymüller-Haapalainen E. Schenkman S. (2011). Three-Dimensional Reconstruction of Trypanosoma Cruzi Epimastigotes and Organelle Distribution Along the Cell Division Cycle: 3D Electron Microscopy of Trypanosoma Cruzi. Cytomet. Part A 79A (7), 538–544. doi: 10.1002/cyto.a.21077 21567937

[B50] Rodríguez M. E. Rizzi M. Caeiro L. D. Masip Y. E. Perrone A. Sánchez D. O.. (2020). Transmigration of Trypanosoma Cruzi Trypomastigotes Through 3D Cultures Resembling a Physiological Environment. Cell. Microbiol. 22 (8), e13207. doi: 10.1111/cmi.13207 32270902

[B51] Romagnoli B. A. A. Picchi G. F. A. Hiraiwa P. M. Borges B. S. Alves L. R. Goldenberg S. (2018). Improvements in the CRISPR/Cas9 System for High Efficiency Gene Disruption in Trypanosoma Cruzi. Acta Trop. 178, 190–195. doi: 10.1016/j.actatropica.2017.11.013 29174293

[B52] Rupp G. Porter M. E. (2003). A Subunit of the Dynein Regulatory Complex in Chlamydomonas Is a Homologue of a Growth Arrest–Specific Gene Product. J. Cell Biol. 162 (1), 47–57. doi: 10.1083/jcb.200303019 12847082 PMC2172716

[B53] Santos C. M. B. dos Ludwig A. Kessler R. L. Rampazzo R. de C. P. Inoue A. H. Krieger M. A. Pavoni D. P. Probst C. M. (2018). Trypanosoma Cruzi Transcriptome During Axenic Epimastigote Growth Curve. Memórias do Instituto Oswaldo Cruz 113 (5). doi: 10.1590/0074-02760170404 PMC5907844 29668769

[B54] Shimogawa M. M. Ray S. S. Kisalu N. Zhang Y. Geng Q. Ozcan A.. (2018). Parasite Motility Is Critical for Virulence of African Trypanosomes. Sci. Rep. 8 (1), 9122. doi: 10.1038/s41598-018-27228-0 29904094 PMC6002391

[B55] Sinclair A. N. Huynh C. T. Sladewski T. E. Zuromski J. L. Ruiz A. E. de Graffenried C. L. (2021). The Trypanosoma Brucei Subpellicular Microtubule Array Is Organized Into Functionally Discrete Subdomains Defined by Microtubule Associated Proteins. PloS Pathog. 17 (5), e1009588. doi: 10.1371/journal.ppat.1009588 34010336 PMC8168904

[B56] Soares Medeiros L. C. South L. Peng D. Bustamante J. M. Wang W. Bunkofske M.. (2017). Rapid, Selection-Free, High-Efficiency Genome Editing in Protozoan Parasites Using CRISPR-Cas9 Ribonucleoproteins. MBio 8 (6), e01788–e01717. doi: 10.1128/mBio.01788-17 29114029 PMC5676044

[B57] Sosa-Hernández E. Ballesteros-Rodea G. Arias-del-Angel J. A. Dévora-Canales D. Manning-Cela R. G. Santana-Solano J.. (2015). Experimental and Mathematical-Modeling Characterization of Trypanosoma Cruzi Epimastigote Motility. PloS One 10 (11), e0142478. doi: 10.1371/journal.pone.0142478 26544863 PMC4636178

[B58] Sun S. Y. Kaelber J. T. Chen M. Dong X. Nematbakhsh Y. Shi J.. (2018). Flagellum Couples Cell Shape to Motility in Trypanosoma Brucei. Proc. Natl. Acad. Sci. 115 (26), E5916–E5925. doi: 10.1073/pnas.1722618115 29891682 PMC6042131

[B59] Sun Y. Wang C. Yuan Y. A. He C. Y. (2012). An Intra-Cellular Membrane Junction Mediated by Flagellum Adhesion Glycoproteins Links Flagellum Biogenesis to Cell Morphogenesis in Trypanosoma Brucei. J. Cell Sci. 126 (2), 520–31. doi: 10.1242/jcs.113621 23178943

[B60] Walker B. J. Wheeler R. J. (2019). High-Speed Multifocal Plane Fluorescence Microscopy for Three-Dimensional Visualisation of Beating Flagella. J. Cell Sci. 132 (16), jcs231795. doi: 10.1242/jcs.231795 PMC6737910 31371486

[B61] Wheeler R. J. (2017). Use of Chiral Cell Shape to Ensure Highly Directional Swimming in Trypanosomes. PLOS Comput. Biol. 13 (1), e1005353. doi: 10.1371/journal.pcbi.1005353 28141804 PMC5308837

[B62] Zhang W.-W. Karmakar S. Gannavaram S. Dey R. Lypaczewski P. Ismail N.. (2020). A Second Generation Leishmanization Vaccine With a Markerless Attenuated Leishmania Major Strain Using CRISPR Gene Editing. Nat. Commun. 11 (1), 3461. doi: 10.1038/s41467-020-17154-z 32651371 PMC7351751

