# Supplementary material for: Trypanin Disruption Affects the Motility and Infectivity of the Protozoan Trypanosoma cruzi
Source: Front Cell Infect Microbiol. 2022 Jan 7;11:807236. doi: 10.3389/fcimb.2021.807236 (PMC8777028; doi:10.3389/fcimb.2021.807236)
Supplement: Supplementary file 1 [file DataSheet_1.pdf]

Supplementary material

Trypanin disruption affects motility and infectivity of the protozoan *Trypanosoma cruzi*

Jose L Saenz-Garcia[1], Beatriz S Borges[2], Normanda Souza-Melo[3,4], Luiz V Machado[1], Juliana S. Miranda[1], Lisandro A Pacheco-Lugo[5], Nilmar S Moretti[3], Richard Wheleer[6], Lia C Soares Medeiros[2], Wanderson D DaRocha[1]*.

S-fig.1

A

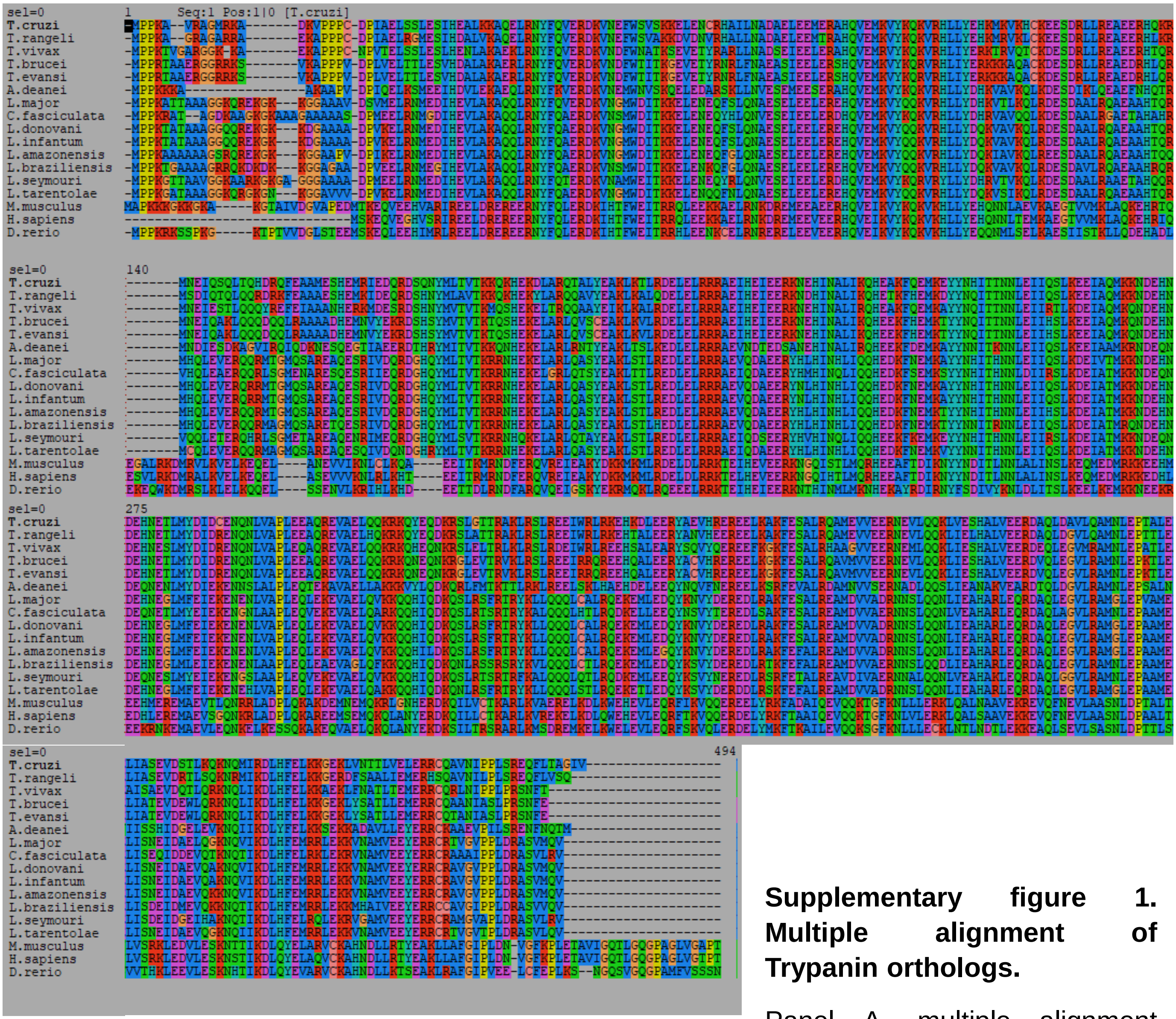

B

ORGANISM	SEQUENCE ID	LENGTH	IDENTITY*
Trypanosoma rangeli SC58	TRSC58_03641	454	84.36
Trypanosoma brucei brucei TREU927	Tb927.10.6350	453	73.17
Trypanosoma vivax Y486	TvY486_1006310	452	73.11
Trypanosoma evansi strain STIB 805	TevSTIB805.10.6710	453	72.95
Angomonas deanei strain Cavalho ATCC PRA-265	ADEAN_000176300	447	60.45
Crithidia fasciculata strain Cf-Cl	CFAC1_250029500	460	57.87
Leishmania amazonensis MHOM/BR/71973/M226	LAMA_000325700	459	57.62
Leishmania donovani BPK282A1	LdBPK_362000.1	459	57.40
Leishmania infantum JPCM5	LINF_360025600	459	57.40
Leishmania major strain Friedlin	LmjF.36.1910	459	57.40
Leishmania braziliensis MHOM/BR/75/M2904	LbrM.35.2110	459	55.85
Leishmania tarentolae Parrot-TarII	LtaP36.1900	459	55.41
Leptomonas seymouri ATCC 30220	Lsey_0075_0140	461	55.41
Mus musculus	NP_061343.2	478	37.47
Homo sapiens	BAH12677.1	453	37.00
Danio rerio	NP_955928.1	475	35.97
Trypanosoma cruzi Dm28c 2018	C4B63_48g99	456	

* Identity data by comparing each ortholog with *T. cruzi* sequence using Clustal Omega

Supplementary figure 1. Multiple alignment of Trypanin orthologs.

Panel A, multiple alignment using Seaview® software. This alignment was used to built the phylogenetic tree presented on Fig1A.

Panel B shows some information of each Trypanin ortholog used of panel A, including its identity with *Tc*Trypanin.

S-fig.2

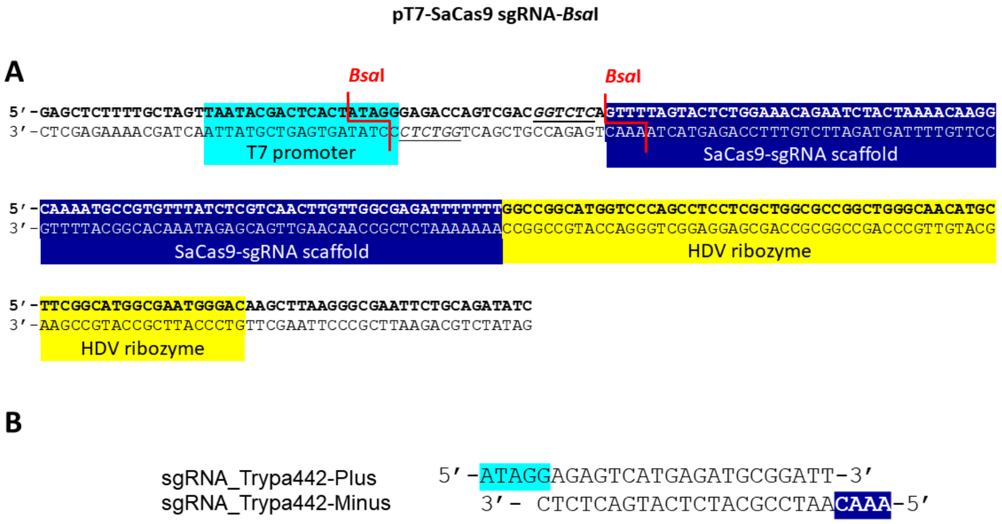

Supplementary figure 2. Scheme of the plasmid pT7-*Sa*Cas9-sgRNA-*Bsa*I. Nucleotide sequence of pT7-SaCas9-sgRNA-BsaI is highlighted to show important sequences for crRNA cloning and *in vitro* transcription. Pale blue: T7 promoter sequence, Blue: Sequence of *Sa*Cas9 scaffold, Yellow: Sequence of HDV ribozyme. The overhangs generated by *BsaI* digestion is highlighted in red.

S-fig.3

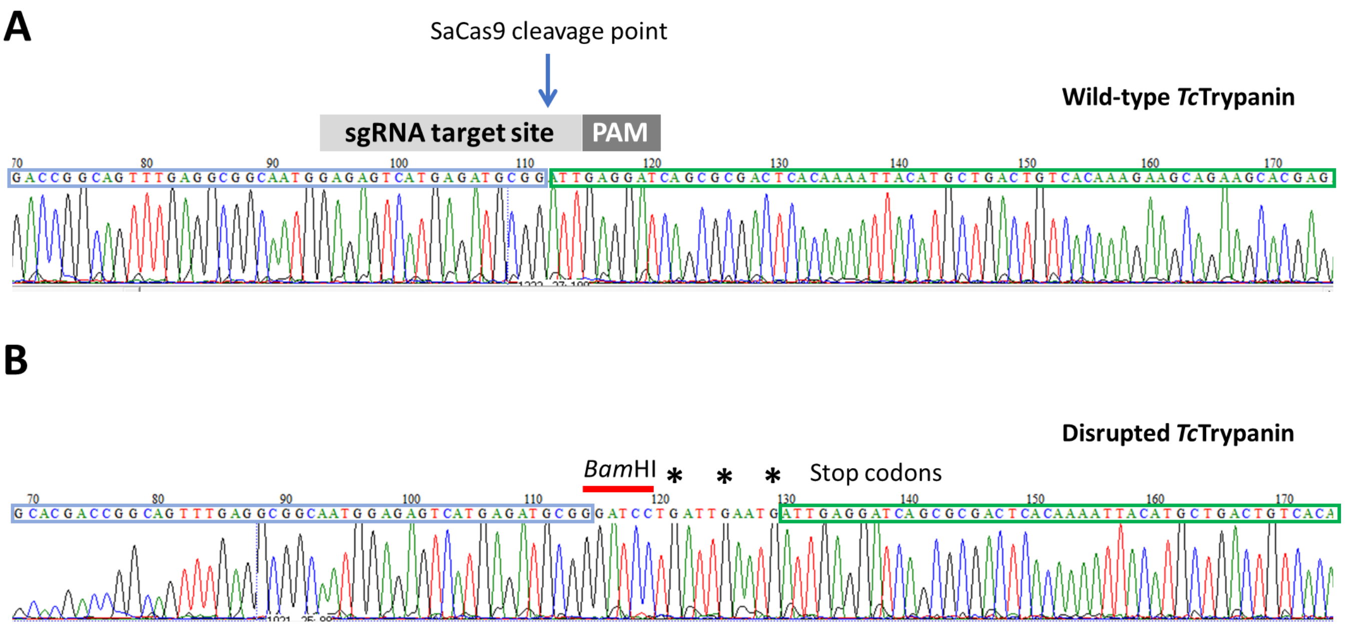

Supplementary figure 3. DNA sequencing of *Tc*Trypanin amplicon from WT and *Tc*Trypanin -/-. Panel A shows the DNA sequencing of *Tc*Trypanin amplicon from WT culture. Blue and green rectangles correspond to upstream and downstream sequences flanking the SaCas9 RNP cleavage site. It is also highlighted the PAM sequence and the crRNA sequence. In B, it shown the DNA sequence (including the electrophoretogram profile of *Tc*Trypanin -/- amplicon. *Bam*HI restriction site and stop codons inserted by donor template recombination are indicated by red line and asterisks, respectively.

S-fig.4

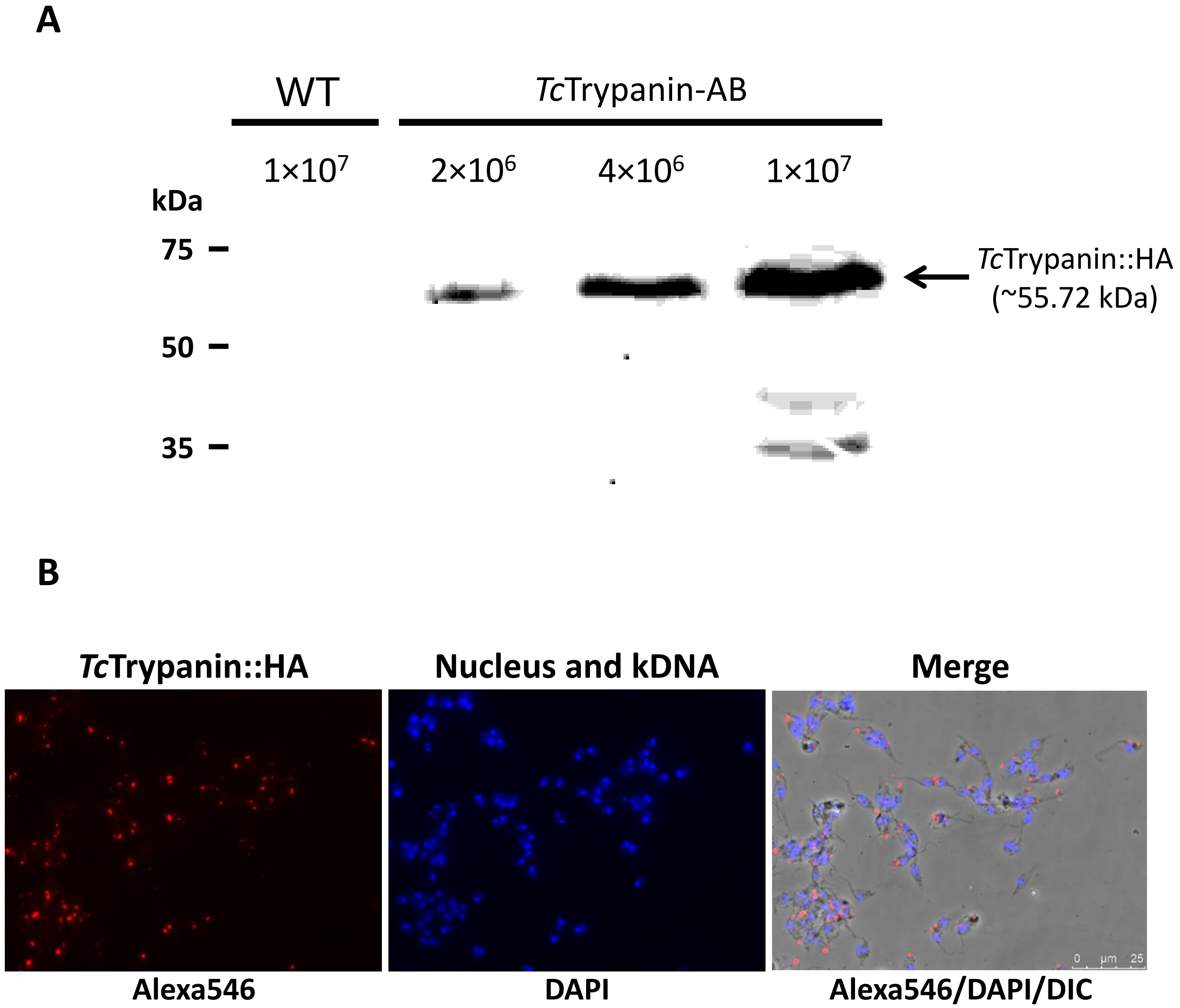

Supplementary figure 4. Western blot and immunofluorescence assay of *Tc*Trypanin -/- overexpressing *Tc*Trypanin::HA. Panel A shows western blot of wild type ($1x10^7$ cells/lane) and *Tc*Trypanin-AB (0.2, 0.4, or $1x10^7$ cells/lane). The theoretical molecular weight of *Tc*Trypanin fused to HA tag is 55.72 kDa. Panel B shows IFA of epimastigotes expressing *Tc*Trypanin::HA. WT cells did not show detectable fluorescence in the Alexa546 channel.

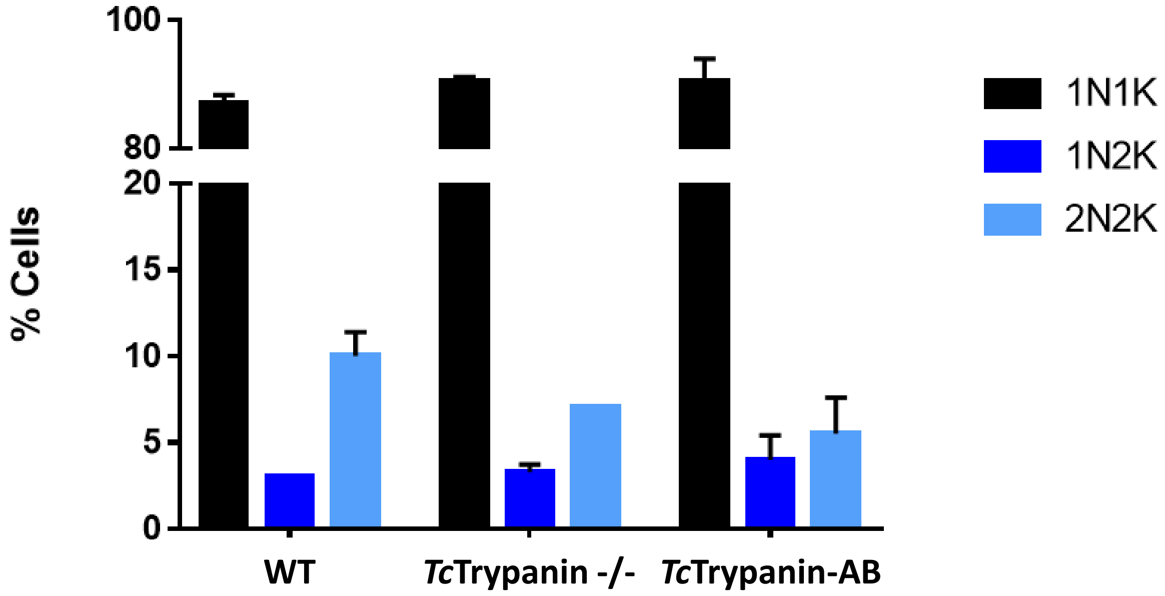

Supplementary figure 5. DAPI analysis. *Tc*Trypanin -/-, WT and *Tc*Trypanin-AB were stained with DAPI, and Nucleus and Kinetoplast were quantified.

S-fig.6

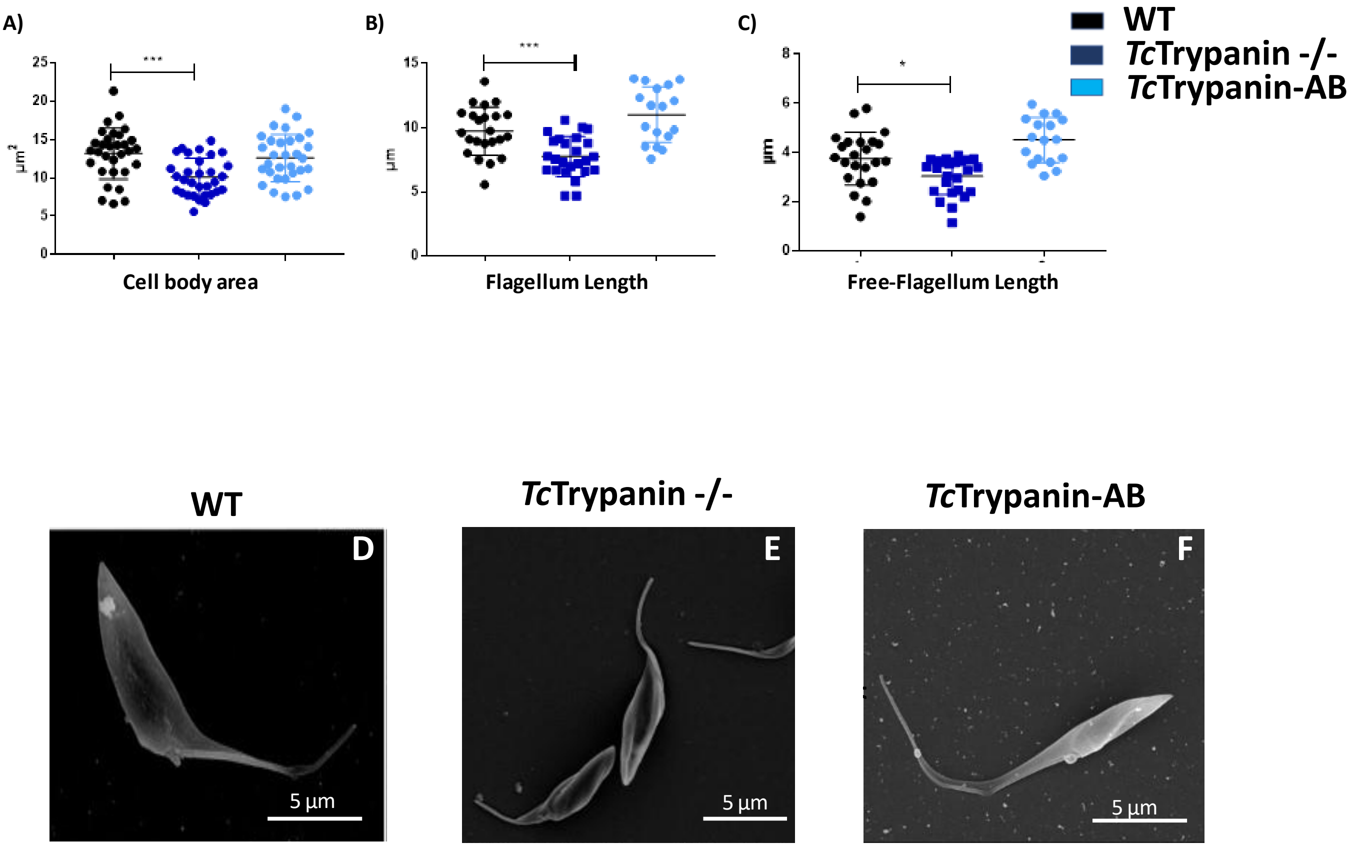

Supplementary figure 6. *Tc*Trypanin disruption interferes with epimastigote morphology. Panels A-C correspond to flagellum length, free-flagellum length and cell body area of WT, *Tc*Trypanin -/-, and *Tc*Trypanin-AB epimastigotes determined by analyzing images from SEM. 20 parasite images were analyzed. The asterisks represent statistically significant difference between WT and *Tc*Trypanin −/− cells (Two-Way Annova, “*” t-test, $p < 0.05$; “***” $p < 0.001$). Panels D-F correspond to SEM images of representative parasites from each culture.

S-fig.7

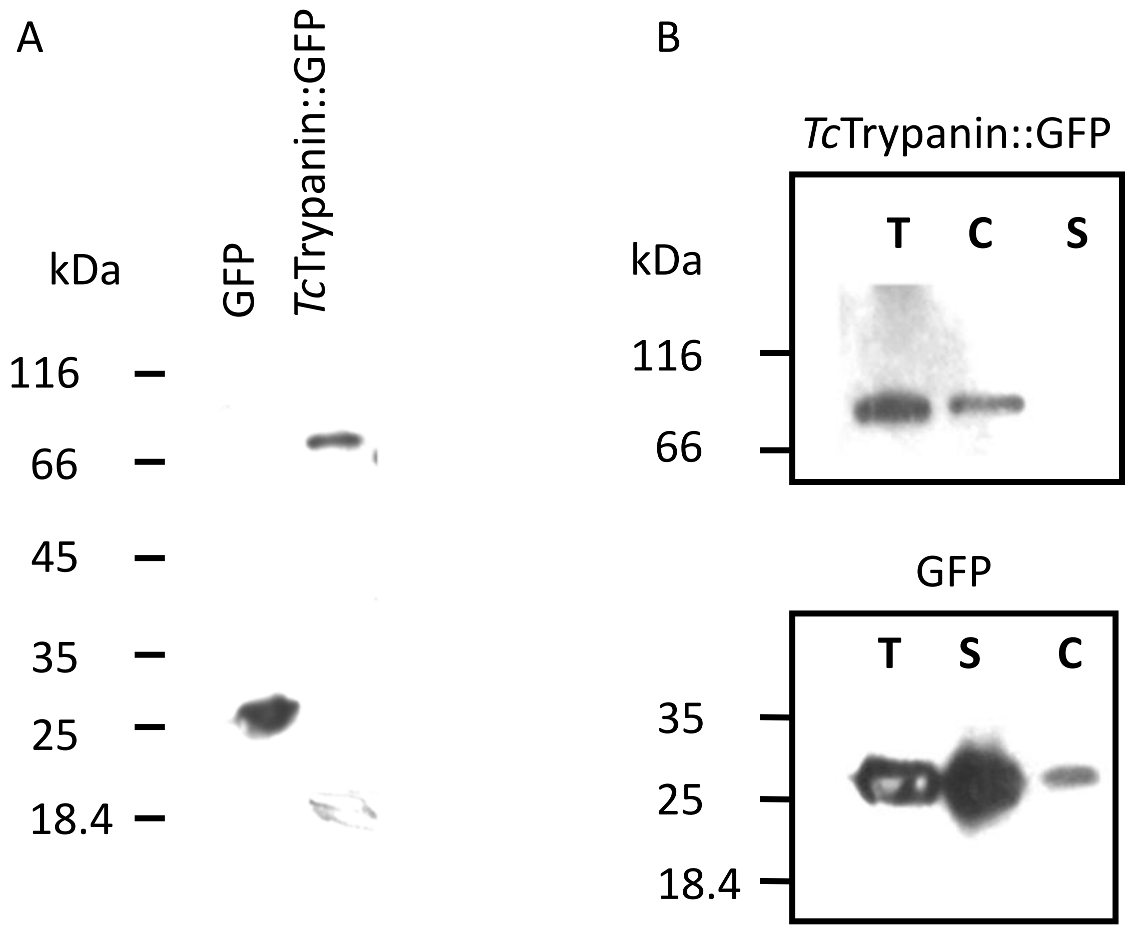

Supplementary figure 7 Western blot of total cell extract and cell fractioning of parasites stably transfected with pTREX-*Tc*Trypanin::GFP. Panel A, Total extract of parasites expressing GFP (expected size = 26.8 kDa) or *Tc*Trypanin::GFP (expected size = 81.8 kDa) incubated with Anti-GFP (rabbit) and Anti-Rabbit conjugated with peroxidase. Panel B, Cytoskeleton was extracted from cells expressing *Tc*Trypanin::GFP or GFP. The fractions were electrophoresed, transferred to membrane, and incubated with Anti-GFP (rabbit) and Anti-Rabbit conjugated with peroxidase. T = Total extract, C = Cytoskeleton preparation (Insoluble fraction) and S Soluble fraction.
